# Chromatin Hubs: A biological and computational outlook

**DOI:** 10.1016/j.csbj.2022.07.002

**Published:** 2022-07-05

**Authors:** Antonio Mora, Xiaowei Huang, Shaurya Jauhari, Qin Jiang, Xuri Li

**Affiliations:** a Joint School of Life Sciences, Guangzhou Medical University and Guangzhou Institutes of Biomedicine and Health (Chinese Academy of Sciences), Guangzhou 511436, PR China; b Affiliated Eye Hospital of Nanjing Medical University, Nanjing 210000, PR China; c State Key Laboratory of Ophthalmology, Zhongshan Ophthalmic Center, Sun Yat-Sen University, and Guangdong Provincial Key Laboratory of Ophthalmology and Visual Science, Guangzhou 510060, PR China

**Keywords:** Chromatin interaction, Chromatin hub, Hi-C, Transcription factory, Nuclear speckles, TAD, LAD, Phase separation

## Abstract

This review discusses our current understanding of chromatin biology and bioinformatics under the unifying concept of “chromatin hubs.” The first part reviews the biology of chromatin hubs, including chromatin–chromatin interaction hubs, chromatin hubs at the nuclear periphery, hubs around macromolecules such as RNA polymerase or lncRNAs, and hubs around nuclear bodies such as the nucleolus or nuclear speckles. The second part reviews existing computational methods, including enhancer–promoter interaction prediction, network analysis, chromatin domain callers, transcription factory predictors, and multi-way interaction analysis. We introduce an integrated model that makes sense of the existing evidence. Understanding chromatin hubs may allow us (i) to explain long-unsolved biological questions such as interaction specificity and redundancy of mechanisms, (ii) to develop more realistic kinetic and functional predictions, and (iii) to explain the etiology of genomic disease.

## Graphical abstract

**Figure:**
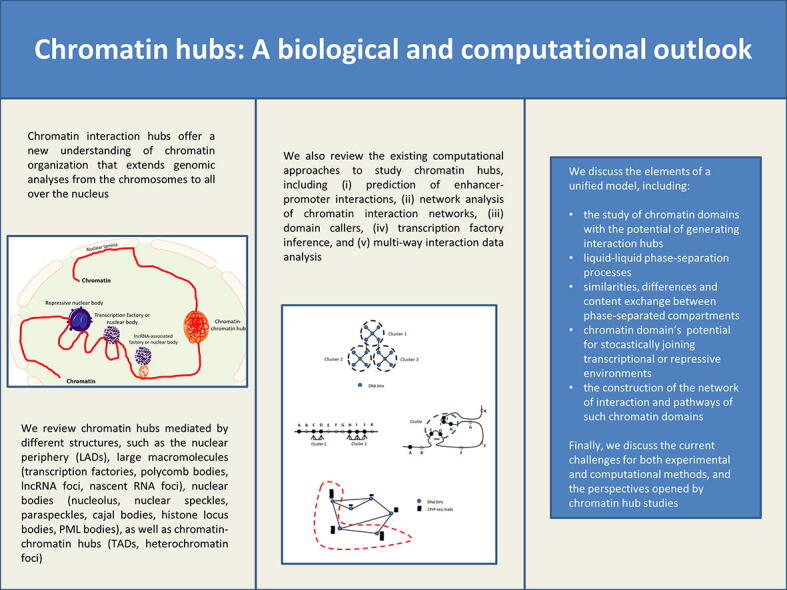

## 1 Introduction

A few years ago, we discussed chromatin interactions' most relevant theoretical and computational aspects in a review that we recommend as a starting point to the readers new to the subject [1]. The review focused on enhancer–promoter interactions through chromatin looping, already recognized as a main mechanism of gene regulation. We discussed data coming from “chromosome conformation capture” (CCC) technologies, a group of technologies that uses cross-linking and sequencing to infer chromatin interactions [2] and includes “high-throughput chromosome conformation capture” (Hi-C) [3], “chromatin interaction analysis by paired-end tag sequencing” (ChIA-PET) [4], and “promoter capture Hi-C” (CHi-C) [5], among others. Those initial studies provided a view of the genome at the chromatin–chromatin interaction scale as a network of short- and long-range interacting loops, as well as a view of the genome at the megabase scale as divided into open and active compartments (“A”) and closed and inactive compartments (“B”). At the chromatin level, we learned that enhancers interact with promoters and these interactions are stabilized by a group of proteins, including CTCF, cohesin, and mediator.

Considerable progress has been made since the time of that review, including the study of “chromatin hubs” or multi-way chromatin interactions (as opposed to pairwise interactions only). The study of such hubs has offered a new understanding of chromatin organization that is not limited to the chromosomes but involves the nuclear periphery, nuclear bodies, and small compartments created around large macromolecules. In this picture, hubs are not random agglomerations of interactions but are compartments with liquid-like properties that possess different functions. Such compartments promote intra-compartmental and discourage extra-compartmental interactions, and help to explain complex phenomena ranging from organelle biogenesis to gene co-regulation and disease. Studying such chromatin hubs is important because most hubs are involved in either the transcription of genes or repression of transcription, which are vital processes to maintaining a cell’s identity.

Consequently, this review extends our previous discussion of single chromatin interactions to hubs of chromatin (their definitions, features, functions, applications, and computational prediction methods). As in the previous review, we will also present the current challenges and perspectives.

## 2 Chromatin hubs: The biological side

### 2.1 There are different types of chromatin hubs

Biologically, a chromatin hub can be defined as a group of chromatin segments that interact directly or mediated by certain proteins, complexes, lncRNAs, or nuclear bodies. Some chromatin hubs are built of chromatin-chromatin interactions only, which are characterized mainly as short loops, with ∼2% of the Hi-C peaks corresponding to loops more than two megabase pairs (Mb) apart [6]. However, many chromatin hubs include chromatin interactions with proteins, lncRNAs, or complex DNA:RNA structures. Computationally, we define a chromatin hub as any group of pairwise interactions that form a module or a connected component in a chromatin interaction network (ChIN), whether the nodes (chromatin regions) are near or distant in a linear view of the genome.

While the biological definition assumes that chromatin hubs physically exist as such at a given time, the computational definition does not consider whether the loops in the hub occur simultaneously or at different times/in different cells. In fact, the chromatin hubs that appear in bulk Hi-C experiments could correspond to an ensemble of all the alternatives in a population of cells, not happening at the same time in any given cell. As an example, MYC, the most central node in most gene-based chromatin interaction networks, interacts with its enhancers in an exclusive and probably stochastic manner. Under the conditions of one study, a given enhancer interacts with MYC in no more than 10% of the cells at any given time [7]. Using terminology borrowed from protein interaction networks, ChINs can display “party hubs” (hubs where all interactions occur simultaneously) and “date hubs” (hubs where interactions are alternative).

From a physical point of view, a new chromatin organization model is gaining acceptance, one in which chromatin hubs form liquid–liquid “phase-separated condensates” that behave as liquid compartments. This image is similar to the current description of membrane-less nuclear bodies, such as the nucleolus. Therefore, the model offers a unified view of nuclear compartmentalization as a series of dynamic “bubbles” able of generation, growth, fusion, fission, and decay. It is known that nuclear bodies are not enclosed by lipid bilayers but, instead, exist in a stable, round-shaped, liquid-like state, similar to oil drops in water, which is also called a “biomolecular condensate” or a “phase-separated” state [8]. These bodies have liquid-like physical properties, such as fusion and fission of droplets, and a high turnover of components. For example, the three compartments of the nucleolus may behave as independent liquid compartments, but the disruption of the nuclear actin leads to their coalescence into a single droplet [9]. Transcription factors (TFs), lncRNAs, and other DNA-binding proteins have also been postulated to form condensates at super-enhancers, in which all the transcription machinery can be concentrated [10], [11]. It has been suggested that some large macromolecules (*e.g.*, lncRNAs or PML proteins) could accumulate at specific chromatin loci and undergo phase separation after reaching a certain saturation concentration, which might be followed by detachment or coalescence of the newly formed condensates [12].

**Fig. 1:**
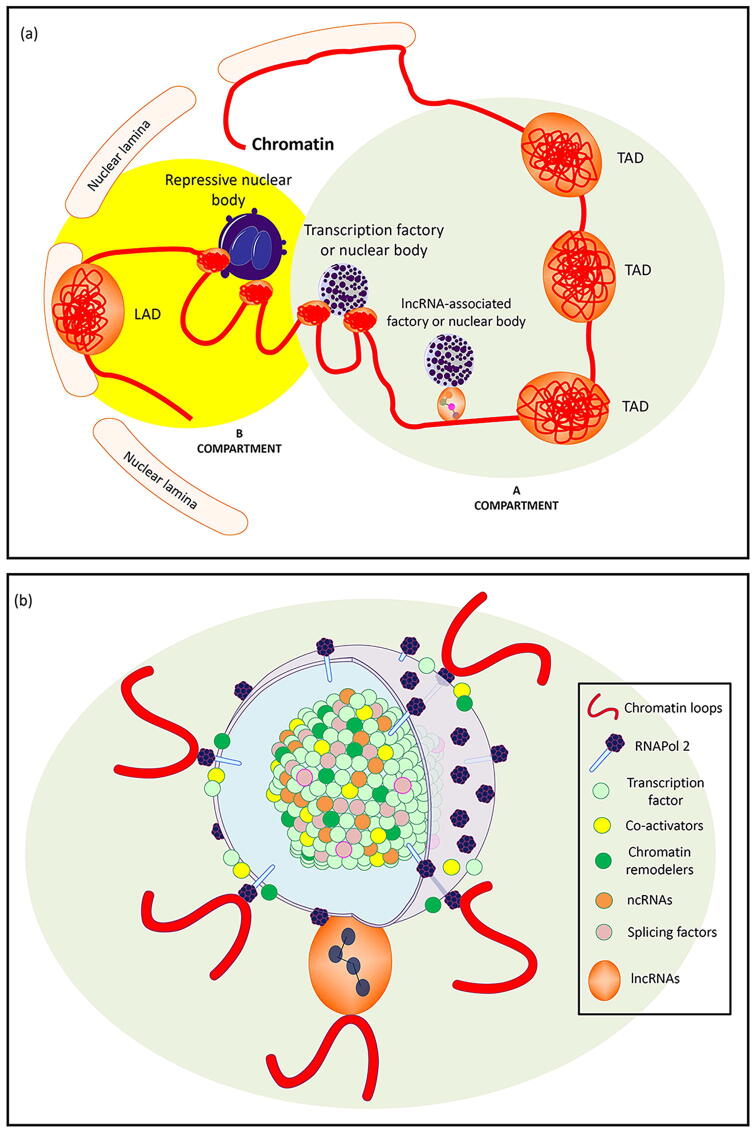
Chromatin hubs and the structures that mediate their interactions. (a) A simplified hub-centered view of the eukaryotic nucleus: Chromatin can loop or hub around (i) repressive environments such as the nuclear lamina, repressive nuclear bodies, or macromolecular structures such as polycomb bodies; (ii) active compartments such as transcription factories, nuclear speckles, and other nuclear bodies; and (iii) chromatin-chromatin interaction-rich environments such as TADs and telomere ends. (b) A sketch view of a transcription-related nuclear body: a phase separated condensate has a core rich in transcription-related proteins and ncRNAs, while chromatin loops are located on its surface. The surface may also contain RNAPol2 molecules (in transcription factories) and spliceosomes (in nuclear speckles). In nuclear speckles and paraspeckles, lncRNAs may bind both the chromosome and the nuclear body.

Our definition of chromatin hubs emphasizes that chromatin interactions can be mediated by certain macromolecules or nuclear bodies. Therefore, we start this review by discussing chromatin interaction hubs in the context of the structures that mediate their interactions. To do so, we have classified chromatin hubs into 18 categories (Table 1, Fig. 1).

**Table 1:** A classification of known types of chromatin hubs.

Hub type	Hub name	Signature 1
Pure chromatin-to-chromatin	Topologically Associating Domains (TADs)	CTCF, cohesin
	HP1/Heterochromatin foci	HP1, Telomeres
Chromatin-to-nuclear periphery	Lamina Associated Domains (LADs)	CTCF, laminA/C, laminB
Chromatin-to-large macromolecules	RNAPol1 Transcription Factories	RNAPol1, rDNA
	RNAPol2 Transcription Factories	RNAPol2, TFs, promoters, enhancers
	RNAPol3 Transcription Factories	RNAPol3, tRNA, housekeeping ncRNAs
	Polycomb bodies	PRC2, H2AK119ub1, H3K27me3
	lncRNA foci	XIST / MALAT1 / NEAT1
	Nascent-RNA foci	eRNAs, pre-mRNAs, R-loops
Chromatin-to-nuclear bodies	Nucleolus / NADs	RNAPol1, UBF, SL-1
	Nuclear speckles	MALAT1, TFs, splicing factors
	Paraspeckles	NEAT1, lincRNA-p21
	Cajal bodies	snRNAs, Coilin, TERC
	Histone locus bodies	Histone genes
	PML bodies	PML, Sp100, p53
Others	Viral DNA RNAPol2 Factories	Viral DNA, RNAPol2
	Senescence-Associated Heterochromatin Foci (SAHF)	HP1, macroH2A, HMGA
	G-quadruplexes (G4s)	G4s, R-loops

1 We have classified 18 of the most studied types of chromatin hubs according to their similarities. “Hub types” refer to the biological structure that acts as a focus for the chromatin loops. “Signatures” are some of the proteins, genes, RNAs, or chromatin structures that can be used to identify such chromatin hub types.

### 2.2 Pure chromatin-to-chromatin hubs

**TADs** are defined as megabase-sized chromatin interaction domains, easy to identify as interaction clusters in a chromatin interaction heatmap (*i.e.*, chromosome ranges with a high density of internal interactions). Their boundaries are enriched for CTCF and cohesin binding sites, as well as some other genes, and, in bulk studies, they have been shown to be highly conserved across species and stable across different cell types [13]. Bulk CCC studies report that the loss of cohesin produces a loss of interactions, but TADs remain intact. In contrast, the loss of CTCF decreases intra-TAD interactions, while increasing inter-TAD interactions [14]. CTCF depletion effects can be observed in ∼80% of TAD boundaries [15]. In addition, studies in mouse embryonic stem cells (mESCs) have shown that CTCF depletion does not disrupt A/B compartments [15]. The number of TADs has been estimated to be over 2000, covering 90% of the genome [16]. CTCF-binding sites have been found both at the boundaries and inside the TADs [6], [16], and highly-interacting domains inside larger interacting domains are observable in heatmaps, suggesting that the definition of TADs is not entirely objective. Recent models suggest that TADs are organized in a hierarchical fashion (*i.e.*, sub-TADs within TADs), with sub-TADs being more variable across cell types [17].

While TADs have been the most studied and reviewed of all chromatin hubs in the last decade, single-cell HiC (sc-HiC) studies have shown that TADs do not appear in single cells as clear as they do in bulk studies. Such findings sparked a debate regarding the existence of TADs in individual cells, particularly whether the absence of TADs is a limitation of current single-cell technologies or TADs from bulk studies are an artificial ensemble of mutually exclusive alternatives from different cells [18]. Imaging studies on single cells have confirmed that CTCF-depleted cells lose the TAD boundaries, while RAD21-depleted cells show decreased intra-TAD interactions, consistent with the above-mentioned roles of CTCF and cohesin. However, the same studies also showed that such “TADs” were variable between individual cells [19]. Multiplexed super-resolution fluorescence *in situ* hybridization imaging showed “TAD-like” regions in single-cells, though these regions have variable borders and do not disappear after cohesin depletion [20]. Recent methods that generate high-resolution and super-long-range datasets in single cells have reported the existence of TADs in ∼75% of cells in mESCs; however, such TADs are not well conserved, with 65% of the detected TADs being highly variable between cells. The authors confirm that the existence of alternative TADs in a population of cells cannot be explained by differences in their cell cycles. Additionally, such “highly variable TADs” cluster in specific “variable TAD regions” [18].

TAD structure has also shown to be highly dynamic. A recent paper has examined the dynamics of the *Fbn2* TAD in mESCs using super-resolution live-cell imaging. The *Fbn2* TAD appears fully un-looped ∼6% of the time, while it is fully looped ∼3% of the time (with a median lifetime of ∼10–30 min). Interestingly, it is in a “partially extruded” state for ∼92% of the time. In such a state, ∼57–61% of the chromatin exists in ∼1–3 cohesin loops while the rest remain un-extruded [21].

Heterochromatin protein 1 (**HP1**) is a fundamental unit of heterochromatin, especially enriched at pericentromeric and telomeric heterochromatin. It has been shown that HP1α in *Drosophila* can nucleate into foci that have liquid-like properties. This suggests that the formation of heterochromatin domains is not mediated by chromatin compaction (an explanation that accounts for the domain isolation but not for domain interactions) but a phase separation mechanism instead [22]. Such properties explain the existence of hubs of telomere ends as telomere droplets from different chromosomes fusing in larger telomeric hubs [23].

### 2.3 Some chromatin hubs occur at the nuclear periphery

It is generally accepted that most active parts of the chromosomes face the nuclear interior, while most inactive parts face the nuclear periphery. **LADs** are chromatin domains consisting of genomic regions that contact the nuclear lamina, and thus, they are essentially repressed. Bulk studies report that human cells have around 1,000–1,500 LADs, with a size of 100 kb–10 Mb, covering around one-third of the genome; however, they are mobile and contact the nuclear lamina in an intermittent manner. LADs include thousands of genes that are either silent or have a very low transcriptional activity (only around 5–10% are highly active). Among other features, they include most gene deserts (*i.e.*, regions larger than 1 Mb without genes) and a subset of telomeric regions; they are enriched for H3K9me2 and H3K9me3, which are characteristic histone marks of heterochromatin, and their position overlaps with the so-called B compartments derived from Hi-C studies. Similar to TADs, LAD borders are enriched with the CTCF protein. TADs and LADs can overlap in multiple cases, but LADs are best correlated to B compartments [24]. Regarding the nuclear lamina proteins associated with LAD positioning, lamins, LBR, and emerin are a group of redundant elements of a scaffolding complex that is bound by LADs [24]. It has also been suggested that LADs contain small regions with enhancers, TSSs, and micro-loops not bound to lamins, which allows them to locally avoid the repressive environment of the nuclear lamina and to interact with active chromatin [25], [26].

LADs have been mapped by DNA adenine methyltransferase identification (DamID) and Chromatin immunoprecipitation sequencing (ChIP-seq) of either laminA or laminB1. Both methods allow identification of LAD sequences. Additionally, fluorescence *in situ* hybridization (FISH) has been used to determine LADs localization [25]. LAD studies have been initially focused on reporting “constitutive LADs” (which are cell-type invariant) and “facultative LADs” (which are cell-type specific) [24]. More recently, single-cell technologies have improved our understanding of LADs. Single-cell DamID of 100 cells showed that some LADs interact with the nuclear lamina in most cells, while others only interact in some of them [27]. According to FISH imaging and 3D modeling, only ∼30% of the LADs identified by sequencing actually relocate to the nuclear periphery. Consequently, the bulk maps (which showed that one-third of the genome is made of LADs) might only be showing the ensemble of all domains that can potentially interact with the nuclear lamina. In addition, it is important to note that there is a fraction of laminA that interacts with active chromatin in the nuclear interior. Such “euchromatin LADs” are possible because of the action of the LAP2α protein, whose depletion relocates all laminA to the nuclear periphery [25], [28]. Differences between allele-specific LADs have also been put forward [29].

### 2.4 Large macromolecules can be hubs for chromatin

**Transcription factories** are chromatin hubs around RNAPol2 and other transcription-related molecules that are postulated to play a central role in transcription.

In the early 1990 s, Jackson *et al.* observed that transcription in HeLa cells occurred at 300–500 specific sites and not across the nucleus, while Iborra *et al.* found two populations of RNAPol2 molecules, one scattered and one organized in clusters that co-localized with transcription [30]. Current studies report between 100 and 8000 transcription factories per nucleus, depending on the experimental method, cell type, and differentiation state. In addition, between 4 and 30 RNAPol2 complexes per factory have been reported, depending on the experimental method and cell type [30].

Factories are currently depicted as polymorphic compartments with a “diameter” of 60–200 nm where RNAPol2 molecules are stationary on the surface and the core is rich in proteins, including TFs, co-activators, chromatin remodelers, histone modifiers, RNA helicases, and splicing factors, among others [31]. Biochemical purification of factories showed enrichment of TFs and nascent RNA, while electron spectroscopic imaging showed that both DNA and nascent-RNA lie on the periphery of a protein-rich core [32]. Electron spectroscopic imaging also suggests that factories are “surrounded by decondensed chromatin fibers,” which originate from more dense chromatin regions [33]. All together suggests that chromatin loops interact with RNAPol2 molecules on the surface of such factories. An example of a factory is the β-globin factory: in mice, the β-globin regulatory locus is located on chr7 around 60 kb upstream of the β-globin gene; after activation, gene and locus interact to form a tissue-specific “active chromatin hub”. Other genes, such as Eraf (which is located ∼25 Mb apart) also join the chromatin hub [34]. Table 2 includes other examples of transcription factories.

**Table 2:** Some reported transcription factories.

Transcription factory	Reference
β-globin	[41]
TH2 cytokines (interleukins)	[42], [43]
Myc	[44]
Oct4	[45]
ER	[46]
Cytochrome *c*	[47], [48]
Znf219 and Sox2	[49]
Hox	[50], [51], [52]
PR	[53]
NFκB	[40]
ERG	[54]
Nanog	[55]
α-globin	[56]
vκ	[57]

The consensus has moved towards the idea that factories are stable structures and not only assemble after transcriptional requirements. Iborra *et al.* showed that the number of transcription factories remains constant over time. Mitchell and Fraser showed that transcription factories exist even if transcription is inhibited [34], while Palstra *et al.* found that most long-range interactions related to the β -globin locus were maintained even though the polymerase was absent [35]. Mitchell and Fraser suggested that the active chromatin hub may form before entering the factory and join or abandon the factory, depending on the presence or absence of transcription [34].

Transcription factories can also be seen as specialized compartments enriched with a given TF, where genes regulated by this TF move to the factory to be transcribed [30]. For example, Schoenfelder *et al.* found the TF Klf1 at ∼40 discrete foci, mostly co-localized with transcription factories, with 59–72% of Klf1-regulated genes (Hbb, Hba, Hmbs, and Epb4.9) being transcribed in the same factories [36]. The existence and role of transcription factories have received considerable experimental support from CCC studies. Dong *et al.* showed that groups of co-expressing genes also co-localize by 3C [37], whereas Papantonis *et al.* [31] using 3C measurements, showed that a rapidly transcribed gene appears to be “permanently bound” to a transcription factory, while a gene that needed ∼75 min for one round of transcription showed chromatin interactions between the factory and different parts of the gene, as if the gene was slowly tracked down by a stationary polymerase.

RNAPol2 transcribes both protein- and miRNA-coding genes [38]. Chen *et al.* used RNAPol2-associated ChIA-PET data to show the existence of chromatin hubs of both protein- and miRNA-coding genes. Genes in such hubs share functionality and are coordinately expressed when they co-localize [39]. A similar finding was reported by Papantonis *et al.* [40]. RNAPol2-factories have also been suggested to be generated through liquid–liquid phase separation, which would allow them to compartmentalize their processes and increase the local concentration of protein machinery by about 1,000-fold [32].

Although RNAPol2 factories are the best studied, the existence of RNAPol1 and RNAPol3 transcription factories has also been confirmed [58]. While RNAPol2 transcribes an extensive repertoire of genes, RNAPol1 is specialized in transcribing ribosomal RNA (rRNA). RNAPol3 is specialized in a group of highly-expressed housekeeping ncRNA genes, including 5S rRNA, transfer RNAs, U6 snRNA, ribonucleases, 7SL RNA, vault RNAs, Y RNAs, SINEs, 7SK RNA, virus-encoded RNAs, as well as several miRNAs and snoRNAs [59]. RNAPol1 and co-factors have been found in factories of 200–500 nm diameter in the fibrillary centers of the nucleolus, while RNAPol3 co-localizes with tRNA genes spatial clusters [58].

**Polycomb bodies** are foci of polycomb group (PcG) proteins that reportedly repress chromatin hubs [60]. PcG proteins are chromatin remodelers known to maintain gene repression. There are two main protein complexes called PRC1 and PRC2, although multiple variants have been identified [61]. PRC2 catalyzes the writing of the H3K27me3 mark on facultative heterochromatin. It has been reported that PRC1 and PRC2 are enriched at promoters and tend to co-localize on the genome to create “polycomb chromatin domains,” which have a repressive nature. Such domains may reach >10 kbp in size and are characterized by high levels of H2AK119ub1, H3K27me3, and PcG occupancy [62]. The mechanisms of PRC1 and PRC2 to repress transcription are independent; however, they co-localize and synergize to repress target genes. Polycomb bodies are then foci of PcG complexes and polycomb chromatin domains that may be separated in a chromosome by several megabases and work as “silencing” compartments. The best-known case is that of the Hox gene cluster, a group of chromatin domains targeted by PcG proteins, which interacts with a large polycomb body. The formation of polycomb bodies has been associated with liquid–liquid phase separation of PRC1 complexes (specifically, the CBX2 and PHC proteins) [63], [64]. Polycomb bodies are also considered to be dynamic structures, with the binding of PcG proteins to the polycomb body being highly dynamic [62], [65].

It has also been suggested that the trithorax group (Trx) chromatin-modifying complexes (which include proteins such as MLL1/MLL2 and SETD1A/SETD1B) can interact with RNAPol2 to inhibit PRC1 and PRC2 and generate “**Trx chromatin domains**” enriched in H3K4me3. One model suggests that chromatin is bistable, switching from repressive PcG to active Trx domains. If transcription activation signals are absent or low, PRC1 and PRC2 can form PcG domains, whereas if they are high, Trx and RNAPol2 antagonize PRC1 and PRC2 and form transcription-permissive Trx domains [62], [66], [67]. It has also been reported that Trx domains are targeted for transcription factories, while PcG domains are targeted for PcG bodies [68].

**lncRNAs** act as miRNA sponges, transcriptional activators or inhibitors, and scaffolds for nuclear organelles, but they can also serve as foci for chromatin hubs. It has been suggested that lncRNA foci also resemble membrane-less organelles. One example is XIST, which mediates X chromosome inactivation and can spread along the condensed X chromosome [60]. NEAT1 and MALAT1 also bind to many sites and often co-localize, but they especially bind to active genes [69]. Moreover, NEAT1 has a protein interaction network that includes proteins related to transcription, splicing, translation, and polyadenylation, among others, which is similar to other chromatin hubs [60]. Werner *et al.* have shown that around 60% of all annotated lncRNAs are chromatin-associated, near active protein-coding regions, and tethered by RNAPol2 [70].

**Nascent RNAs** have been reported to form clouds over regulatory DNA elements, which link distant promoters and enhancers and generate “nascent-RNA-associated transcription hubs” [23], [71], [72]. Such hubs contain, on average, around 4 active promoters, 20 typical enhancers, and 1–2 super-enhancers. Some examples of nascent-RNAs involved in transcription regulation include (i) promoter upstream transcripts (PROMPTs), which participate in the recruitment of TFs and chromatin remodelers; (ii) enhancer RNAs (eRNAs), the production of which is correlated to enhancer activity; and (iii) pre-mRNAs, for whom a gene regulatory function (beyond being a mere intermediate step in the mRNA production) has started to be discussed [23]. Nascent RNAs are retained at their site of transcription by mechanisms such as “R-loops”: RNA:DNA hybrids between the nascent RNA and the template strand. Such structures have been considered both a source of genomic instability (making some regions more sensitive to DNA damage) and regulators of gene expression (R-loops in the promoter may lead to transcriptional repression by RNAPol2 pausing). Another retention mechanism is the interaction of PRC2 with nascent RNAs, such as pre-mRNAs [23]. Other components of the nascent RNA interactome have also been published [73].

### 2.5 Chromatin hubs exist around nuclear bodies

Several membrane-less organelles or “nuclear bodies” interact with chromatin, and therefore, may also serve as foci for chromatin hubs. These include the nucleolus (for ribosomal DNA), nuclear speckles and paraspeckles (for pre-mRNAs from diverse genes), Cajal bodies (for snRNAs), histone locus bodies (for histone mRNAs), and PML bodies (for both euchromatic and heterochromatic DNA).

The entire **nucleolus**, rather than only RNAPol1 factories, can be considered a chromatin hub, as multiple segments of chromatin interact with the nucleolar periphery. Such chromatin regions are enriched on repeats of the ribosomal gene (rDNA) and have been called “perinucleolar chromatin,” “nucleolar organizing regions,” or “nucleolus-associated chromatin domains” (NADs) [74], [75]. Similar to LADs, NADs are B compartments with low gene density and expression level [25]. Two-thirds of NADs reportedly overlap with LADs in human fibroblasts, meaning one-third of NADs are perinucleolus-specific [76]. Also, NADs display more euchromatin than heterochromatin, suggesting that they are less repressive than LADs [74]. Lamins have been reported at the nucleolar periphery, but it is not clear if they tether NADs in the same way as LADs at the nuclear periphery [25]. One biogenesis model suggests that small nucleoli assemble around each NAD and form compartments that recruit the necessary machinery for rRNA transcription, rRNA processing, and ribosome assembly, including RNAPol1, the DNA-binding protein UBF, and the SL-1 complex for rRNA transcription, as well as small nucleolar ribonucleoproteins (snoRNPs), for post-transcriptional modifications. Subsequently, all the small nucleoli seem to interact and condensate in a single large nucleolus [75]. It is important to note that the nucleolus is a highly dynamic structure, with RNAPol1 components, for example, being continuously exchanged and only transiently localized at nucleoli [74].

**Nuclear speckles** (also known as splicing speckles, SC-35 domains, or interchromatin granule clusters) were previously considered to be just a place for the storage of splicing factors. Modern techniques have elucidated their role in gene expression, since they contain numerous proteins related to epigenetic regulation, chromatin organization, TFs, and ncRNAs. The existence of chromatin-nuclear speckle hubs has been proposed, in which such hubs might coordinate all the gene expression regulation steps in a way similar to a transcription factory [77]. Similar to most nuclear bodies, splicing speckles are a liquid-like entity with a density and protein concentration slightly higher than the surrounding nucleoplasm. A human nucleus contains 20–50 speckles, each with a diameter of several micrometers, composed of several spots measuring 20–25 nm. The proteins discovered in the speckles include TFs, splicing factors, chromatin remodeling proteins, protein kinases, PI signaling proteins, nucleoskeletal organization proteins, ubiquitination and SUMOylation proteins, and ncRNAs (such as spliceosomal snRNA, 7SK RNA, and NEAT2 lncRNA) [77]. Co-expressed genes are known to co-localize at nuclear speckles [78], as has been observed for both α- and β-globin in human erythroblast cells, as well as adipogenic genes in porcine adipocytes [79]. Khanna *et al.* reported the repositioning of the HSP70 gene to nuclear speckles after heat shock, followed by their transcription [80]. Nuclear speckles co-localize with MALAT1, a lncRNA that interacts with active promoters. MALAT1 has been suggested to act as a link between chromatin and nuclear speckles [69].

**Paraspeckles** are formed near nuclear speckles [23] and their chromatin interactions are mediated by NEAT1 (a lncRNA that binds to actively transcribed genes). NEAT1 has been reported to induce paraspeckle formation through phase separation [81]. Paraspeckle/NEAT1 chromatin hubs are associated with both histone-modifying and nucleosome-remodeling enzymes. Some studies suggest that paraspeckle/NEAT1 regulate transcription, as NEAT1 regulates histone marks that stimulate transcription, while members of the SWI/SNF complex co-localize with paraspeckles. However, other studies suggest that paraspeckle/NEAT1 regulate repression, as NEAT1 interacts with members of PRC2. Paraspeckles also interact with lincRNA-p21 [60], [69].

**Cajal bodies** are nuclear compartments located at the interface between chromosome territories, near chromatin domains enriched on small nuclear RNA (snRNA) genes or small nucleolar RNA (snoRNA) genes [82]. They have been linked to the generation and recycling of snRNAs, which are required for pre-mRNA splicing [23]. Cajal bodies can be identified by the coilin protein, Cajal body-specific RNAs called scaRNAs, and splicing small nuclear ribonucleoproteins (snRNPs) [83]. As with other nuclear bodies, they have been shown to interact with a lncRNA, telomerase RNA component (TERC), and to shuttle between the chromatin and interchromatin space [60]. **Histone locus bodies** are similar to Cajal bodies, but they have been differentiated because they interact with chromatin domains enriched on the genes that code for histones, and they contain factors needed to process histone pre-mRNAs [83]. They have also been shown to interact with the Y3/Y3** ncRNA [60].

Finally, promyelocytic leukemia nuclear bodies (**PML bodies**, also known as ND10) are compartments with a diameter of approximately 0.1–1 μm that are involved in H3.3 chromatin assembly, SUMOylation, sequestration of TFs, senescence, and antiviral defense. Around 5–30 PML bodies exist in a cell, depending on cell type and cell cycle phase, forming at regions of high transcriptional activity or near telomeric DNA. They are mainly characterized by the PML protein, which is located at their periphery, enclosing a core filled with both constitutive and transient proteins, especially chromatin-related factors, such as histone H3.3 and histone modifiers (associated with histone methylation, demethylation, acetylation, and deacetylation) [12].

Chromatin fibers have been detected at the periphery of PML bodies [84]. Some publications report nascent RNA inside PML bodies, while others report the accumulation of nascent RNA in their vicinity [85]. Viral DNA has also been detected. In some cases, such as the herpes simplex virus 1 (HSV-1), human papilloma virus 11, Epstein–Barr virus, and bovine papillomavirus, viruses might use the PML bodies as sites for replication/transcription; in some other cases, such as latent HIV proviruses, PML bodies were found to promote transcriptional silencing. HSV-1 is a special case where the viral genome does not integrate into the host genome but remains as an extrachromosomal replicating dsDNA; here, PML bodies may participate in the “chromatinization” of the viral genome by providing the required histone variants and marks [12]. PML bodies have been associated with transcriptionally active chromatin domains, building hubs through chromatin-interacting proteins such as PML, Sp100, and p53 [60]. Chromatin domains known to co-localize with PMLs include the major histocompatibility complex (MHC), TP53, Oct3/4, and DDTI4 loci, among others. PMLs could also play a role in the organization of repressive domains, as they concentrate both SETDB1, which deposits the H3K9me3 mark, and HP1 α, which allows heterochromatin compartment formation by phase separation. There have also been reports of associations with pericentromeric and telomeric heterochromatin under specific pathologies and cell cycle stages. PML bodies that co-localize with telomeres are more common in stem cells and are mostly lost after differentiation; however, some telomeric PML bodies can be found in cells with shortened or damaged telomeres, suggesting that PML bodies might favor telomere elongation or renewal [12].

### 2.6 There are other types of chromatin hubs

Novel types of chromatin hubs are being constantly described. For example, an **RNAPol2 transcription factory with viral DNA** (instead of its host DNA). It has been reported that DNA of the HSV-1 virus can form a replication compartment around RNAPol2 foci [86].

In senescent cells and cells with “laminopathies” (diseases due to lamin protein mutations), it has also been reported that the lamina is disrupted and LADs re-localize away from the nuclear periphery or the nucleolus, accompanied by the formation of **senescence-associated heterochromatin foci (SAHF)** [87]. In senescent cells, SAHF contain facultative heterochromatin and heterochromatin-forming proteins, such as HP1, the histone variant macroH2A, and HMGA [88].

Additionally, DNA domains belonging to **G-quadruplexes** (**G4s**) could be considered either chromatin hubs or a part of a chromatin hub. G4s are DNA secondary structures consisting of stacks of guanine tetrads (four guanine bases associated in a planar structure) in Guanine-rich regions. Although they were initially reported as transcriptional repressors at oncogene promoters, current evidence suggests that G4s are mainly transcriptional enhancers located at the promoters of transcriptionally active genes [89]. Evidence includes G4s being identified as hubs for TF binding [90] and being reported to trigger RNA phase separation [91]. Finally, a recent paper has shown that G4s co-localize with transcription factories and nuclear speckles to a considerable extent, and with PML bodies and Cajal bodies to a lesser extent [92].

### 2.7 A summary of new experimental technologies

In recent years, novel experimental technologies have revolutionized the study of chromatin interactions, the most popular development being “single-cell HiC” [93], [94], [95], [96], [97], [98], [99], a method in which the Hi-C protocol is performed in individual nuclei and not after the nuclear lysis of multiple cells. This method has shown that interactions are sparse in individual cells and the properties of bulk Hi-C interaction maps resemble those of the pool of single cells [93].

Several methodologies to identify RNA–chromatin interactions have also appeared. Some of them are valid for one RNA at a time, such as “chromatin isolation by RNA purification” (ChIRP), “capture hybridization analysis of RNA targets” (CHART), and RNA antisense purification (RAP). A few others can be applied to evaluate RNA–chromatin interactions across all RNAs, such as “mapping RNA–genome interactions” (MARGI), “global RNA interaction with DNA sequencing” (GRID-seq), “chromatin-associated RNA sequencing” (ChAR-seq), “*in situ* MARGI” (iMARGI), and “RNA and DNA interacting complexes ligated sequencing” (RADICL-seq) [23], [100]. There are also novel techniques for nuclear body studies. Two examples for studying PML body specific chromatin are: immuno-TRAP (for a specific chromatin locus) [101] and ALaP-Seq (for the whole genome) [102]. Also, a few methodologies have been proposed for mapping R-loops, such as MapR [103].

We want to highlight recent experimental techniques that go beyond pairwise interactions and detect multiple-loci or multi-way interactions. Some authors argue that the “proximity ligation” approach is not useful for regions too far apart to be directly ligated (*e.g.*, to identify interactions between chromatin regions around a nuclear body); therefore, ligation-free methods have been developed. Other authors see a problem in that 3C-based protocols generate large concatemers that are trimmed in order to be sequenced; therefore, they focus on keeping the full concatemers and using long-read sequencing to detect multi-way interactions. In summary, current multi-way interaction detection methods include ligation-based methods (C-walks [104], Tri-C [105], MC-4C [106], and Pore-C [107]) *versus* ligation-free methods (GAM [108], SPRITE [109], ChIA-Drop [110], and sc-SPRITE [18]), bulk methods (the majority) *versus* single-cell methods (multiplexed super-resolution FISH [20] and sc-SPRITE [18]), Illumina short-read sequencing (the majority) *versus* long-read sequencing (MC-4C [106] and Pore-C [107]), and targeted/high-resolution methods (Tri-C [105] and MC-4C [106]) *versus* high-throughput/low-resolution (the majority). These methods are further reviewed in Supplementary Material 1.

## 3 Chromatin hubs: The computational side

**Fig. 2:**
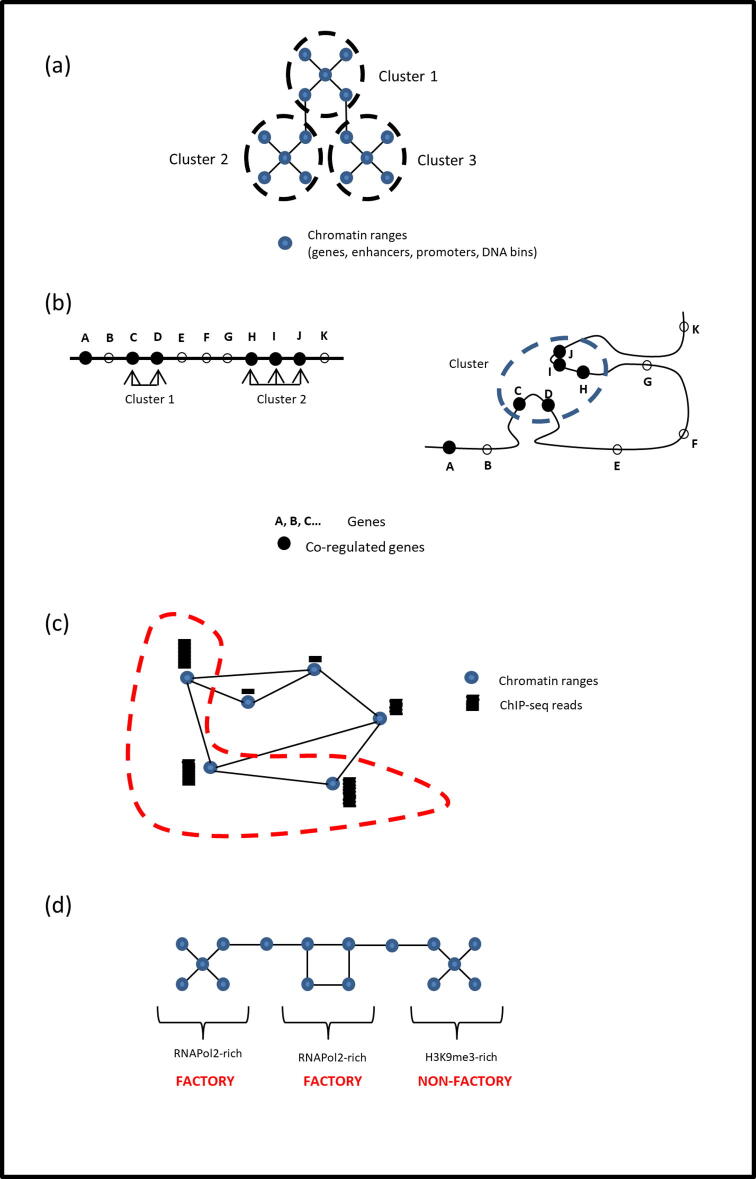
Example of some approaches to computational prediction of chromatin hubs. Multiple approaches for chromatin hub prediction are possible. (a) Simple clustering of a chromatin interaction network. (b) Finding significance of spatial clustering *versus* linear clustering. (c) Superposing a ChIP-seq track onto a chromatin interaction network and detecting regions where the signal is significantly high. (d) Assigning weights to edges according to epigenetic marks, performing weighted clustering, and then using a classifier.

Computational studies of chromatin hubs are a few steps behind the current biological knowledge. However, progress has been made on multiple fronts, including: (i) prediction of enhancer–promoter interactions, using either machine learning models based on different genomic features or natural language processing methods applied to the DNA sequence; (ii) both traditional and novel network analyses of chromatin interaction networks; (iii) development of different types of domain callers to call TADs, LADs, and other domains from genomic data; (iv) various chromatin hub and, specifically, transcription factory inference methods based on statistical comparisons; and (v) novel methods of multi-way interaction data analysis (Table 3; Fig. 2).

**Table 3:** Summary of computational methods to study chromatin hubs.

Category		Methods/Tools reviewed	Reference
Enhancer–promoter interaction prediction		Epigenomics-based methods: FANTOM5, PreSTIGE, IM-PET, RIPPLE, TargetFinder, and JEME	[111], [112], [113], [114], [115]*, and*[116]
		Sequence-based methods: PEP, EP2vec, SPEID, CNN with TL, and SEPT.	[117], [118], [119], [120]*, and*[121]
Network analysis of interaction networks		Standard chromatin interaction network analysis: Sandhu *et al.*, Li *et al.*, Chen *et al.*, Thibodeau *et al.*, and Li *et al.*	[122], [123], [39], [124]*, and*[71]
		Promoter- or enhancer- enriched standard interaction network analysis: Schoenfelder *et al.* and Madsen *et al.*	[125], [126]
		Multi-scale network analysis	[127]
		Graphlet approach	[128]
		Detection of chromatin hubs around disease-related SNPs	[129]
Calling special chromatin domains such as TADs, LADs, NADs, etc.		TAD callers: TopDom, HiCseg, CaTCH, CHDF, and IC-Finder	[130], [131], [132], [133]*, and*[134]
		LAD callers: EDD and LADetector	[135], [136]
R-Loop / G-quadruplex prediction		R-Loop Tracker	[137]
		Intramolecular G4 predictors: Quad-Parser, QGRS Mapper, G4P Calculator, QuadBase, and G4Hunter	[138], [139], [140], [141]*, and*[142]
		Intermolecular G4 predictors: ddiQFP and Allquads	[143], [144]
Chromatin hub / Transcription factory prediction		Comparing co-regulated clusters to background clusters	[145]
		Comparing chromatin hubs in a population: “VCMs” (Intra-TAD and inter-individual variation modules), “Regulatory communities” (Population-conserved 3D chromatin hubs), and “CMINT” (Dynamic changes of chromatin modules)	[146], [147]*, and*[148]
		Overlap of chromatin hubs with significantly high ChIP-seq peaks: “Functional 3D hot-spots”, Belyaeva *et al.*, and Stevens *et al.*	[149], [150]*, and*[96]
		EpiTensor (modeling method detecting “interaction hotspots”)	[151]
Multi-way interaction data analysis		Multi-way interaction callers: SLICE, MIA-Sig, Pore-C-pipeline, and MATCHA	[108], [152], [107]*, and*[153]
		Multi-way interaction prediction based on pairwise interactions	[154]

### 3.1 Prediction of enhancer–promoter interactions

Enhancer–promoter interaction prediction is different to the computational prediction of chromatin hubs; however, predicted interactions can be aggregated into networks and subjected to clustering, as a way of predicting enhancer–promoter hubs.

In our previous review [1], we discussed a few methods based on correlations to single epigenetic marks [155], [156], [157], [158], as well as others based on more sophisticated machine learning classifiers [111], [112], [113], [114]. In both cases, the underlying assumption was that some genomic features (or combination of features) in the 1D genome, such as TF binding, chromatin-binding protein motifs, and histone marks, contain all the necessary information to predict 3D structures, such as chromatin loops. Since the time of our previous review, several new methods have appeared. Here, we review TargetFinder, which we consider to be state-of-the-art in terms of methodology and results, and JEME, which has been used to create a valuable map of chromatin interaction networks.

**TargetFinder** is a method that applies ensembles of boosted decision trees to hundreds of genomic features in six ENCODE cell lines. The method is as follows: enhancers and promoters are identified using segmentation data from ENCODE, while enhancer–promoter interactions are extracted from Hi-C data (dataset from Rao *et al.* [6]). The model is fed with pairs of enhancers and promoters (annotated as interacting or non-interacting), as well as genomic features for each pair, including (i) open chromatin, (ii) DNA methylation, (iii) gene expression, and (iv) ChIP-seq peaks for TFs, architectural proteins, and modified histones. Using recursive feature elimination, the authors identified the minimal subset of genomic features that accurately predicts the interaction pairs for all cell lines. Features were ranked according to its importance, with 16 found to be enough for near-optimal performance.

According to TargetFinder results, the models and ranks of features are different for different cell lines; however, some predictors performed consistently well across cell lines, including CAGE (e-RNA) data, activation-associated (H2AZ, H3K27ac, H3K9ac, H3K4me1, H3K4me3, and H3K4me2) and elongation-associated marks (H3K36me3, H3K79me2), structural proteins [*e.g.* CTCF, cohesin (SMC3 and RAD21), and ZNF384], open chromatin (DNase-seq), and DNA methylation. Some highly–ranked proteins, which are not commonly reported as predictors, included CUX1; SRF; SUPT20H; EBF1; MAX; TFAP2C; chromatin looping related proteins SP1, SPI1 (PU.1), HCFC1, and TBP; and histone modifiers KDM1A and RCOR1. CAGE (e-RNA) was consistently the best predictor in both multivariate (with all 16 features) and univariate (with one feature) models, suggesting that, in case we can only obtain data from one feature, e-RNA should be the chosen one. SUMOylation, a post-translational modification not included in ENCODE or Roadmap Epigenomics at the date of the publication, was found to be another good predictor. RNAPol2, commonly included in software for enhancer–promoter prediction, was not predictive on its own; however, elongation-associated histone marks proved to be more indicative of promoter activity. Additionally, PRC2 and heterochromatin marks were associated with non-interacting pairs [115].

A method called “Joint Effect of Multiple Enhancers” (**JEME**) has been used to predict enhancer–promoter interaction networks for 935 human cells and tissues [116]. The authors built a minimal model based on enhancer features (eRNAs and three histone modifications) and gene expression levels. Enhancer features and gene expression levels are first collected (935 human primary cell types and cell lines, 127 from Roadmap Epigenomics and 808 from FANTOM5). Then, enhancers within 1 Mb of each TSS are considered as its potential enhancers and multiple regression of all enhancers across all samples is performed. The authors introduced a supplementary website (https://yiplab.cse.cuhk.edu.hk/jeme/), including the 935 networks and the software to generate them, which can be retrained if more data becomes available. In comparing enhancer–promoter networks between samples, the study found that biologically-related samples produced similar networks [116].

“Sequence-based methods” have emerged as a novel approach to enhancer–promoter prediction. Such methods assume that a DNA sequence contains all the information needed to predict interactions, using NLP techniques to analyze the DNA sequence (*i.e.*, the enhancer and promoter sequences), instead of information from binding proteins or epigenetic marks. NLP techniques are widely used in websites and apps, where they transform human language sentences into vectors susceptible to machine learning modeling. Sequence-based methods include PEP [117], EP2vec [118], SPEID [119], CNN with TL [120], and SEPT [121]. A brief review of such methods can be found in Supplementary Material 1. It remains to be seen whether sequence-based methods, although using state-of-the-art machine learning, can deliver useful insights while ignoring all transcriptional and epigenetic information.

Another novel approach is followed by Chromatin Interaction Site Detector (**CISD**) and CISD-based loop predictor (CISD_loop) [159]. The authors combine low-resolution Hi-C data and nucleosome information from MNase-seq data to generate high-resolution chromatin interaction networks. The method is based on the observation that strong periodic patterns exist in the nucleosome arrangements flanking chromatin interaction sites; moreover, for allele-specific chromatin interactions, such patterns differ between the interacting and non-interacting alleles.

### 3.2 Network analysis of chromatin interaction networks

Network analysis has been applied to ChINs with mainly descriptive purposes, although some predictive studies have also been conducted. In such networks, nodes represent chromatin regions, such as genes, enhancers, promoters, and DNA segments (bins), while edges represent chromatin interactions.

In 2012, Sandhu *et al.* characterized chromatin hubs and communities using a conventional graph theory approach [122]. They built a ChIN based on RNAPol2-associated ChIA-PET datasets for K562 and MCF7 cells. The result was ∼10,000 connected components, with 40% of the nodes in a giant component that followed a scale-free-like degree distribution, rich-club structure, and contained 1,173 communities. Intra-community interactions happened to be *cis-* (in the same chromosome), while inter-community interactions were either inter-chromosomal or super-long range. In addition, communities were largely conserved between K562 and MCF7 (71% of communities showed >75% overlap). In the network, nodes containing SNPs had a low degree, while hubs lacked SNPs. Finally, 62% of rich-club genes were annotated as lethal in mice.

Similar analyses have been performed on different systems or using different technologies. For example, Li *et al*. used RNAPol2 ChIA-PET data to build a chromatin interaction network in five human cell lines (MCF7, K562, HeLa, HCT116, and NB4), which contained 5% of promoter-to-gene-body, 20% of enhancer–enhancer, 33% of enhancer–promoter, and 42% of promoter–promoter interactions. The network displayed hubs of promoters (called “multigene interaction complexes”), which included multiple promoter–promoter and enhancer–promoter interactions and sometimes interacted with similar hubs in the same or in another chromosome to generate “higher-order multigene interaction complexes.” The study identified 1,328 such hubs containing 11,723 genes (on average, 8.8 genes per hub), which the authors presented as evidence of promoter–promoter interactions being widespread and evidence of a physical mechanism that explains the combinatorial complexity of transcriptional regulation [123]. Chen *et al*. built chromatin interaction networks for “miRNA gene–miRNA gene”, “miRNA gene–target”, and “miRNA gene–protein-coding gene” interactions, using RNAPol2-associated ChIA-PET data in K562 and MCF7 cell lines. They showed that hubs of miRNA genes frequently belong to the same family and disease, while hubs of co-expressed and co-localized miRNA and protein-coding genes suggest that miRNA is also generated in transcription factories. Some hubs were common to both cell types, while others were cell-specific [39].

Schoenfelder *et al.* built a chromatin interaction network for mESCs and fetal liver cells (FLCs) using CHi-C. The CHi-C method enriches Hi-C libraries for promoters and claims a >10-fold enrichment of reads involving promoters. More than 59% of the promoter–genome interactions, and more than 73% of the promoter–promoter interactions were cell-specific; 66.6% of active promoters interacted with the nearest enhancer. Most promoter–genome interactions occurred within TADs, while only a small fraction occurred in LADs. Notably, active promoters showed more long-range interactions than inactive promoters [125]. Similarly, Madsen *et al.* generated a ChIN of enhancers and promoters in mesenchymal stem cells using “enhancer capture Hi-C” (ECHi-C), a method that enriches for enhancers. After clustering, they identified 5,238 enhancer-containing communities, 2,842 of which did not contain promoters. The other 2,396 chromatin hubs (called “gene regulatory communities”) were classified into two groups: ∼61% were “regular communities” and ∼39% were “highly interconnected enhancer communities” (defined as those communities with at least one highly interconnected enhancer and one promoter) [126].

Thibodeau *et al.* built ChINs in three cell lines (K562, MCF7, and GM12878) and analyzed four types of genomic elements: promoters, enhancers, broad H3K27ac marks (also known as “super-enhancers”), and broad H3K4me3 marks (also known as “broad domains”). The authors reported different connectivity patterns for broad domains and super-enhancers that were conserved across cell types. The number of interactions between such elements was higher than theoretically expected, *e.g.*, from broad domains to all other nodes (2.9 times higher than expected), super-enhancers to broad domains (2.7–5.5 times), and super-enhancer to super-enhancer (2.7–5.0 times). Broad domains were more connected than normal promoters, while super-enhancers were more connected than normal enhancers. Finally, the authors built a support vector machine that allowed the discrimination of broad domains from regular promoters and super-enhancers from regular enhancers by using both network connectivity metrics and genomic datasets as features [124].

It is also important to note a recent publication of the RNA–chromatin interactome of the human chromosome 11 for breast cancer cells, which includes a network of nascent-RNA hubs [71].

Other network studies have introduced more complex analyses. Babaei *et al*. built a multi-scale ChIN using data from the Allen Mouse Brain Atlas to assess whether multi-scale chromatin interactions performed better than single-resolution chromatin interactions at predicting co-expression patterns. The multi-scale network included large-scale interactions (between chromatin compartments), medium-scale interactions (between genes), and small-scale interactions (between TSSs of genes). The authors computed scale-aware versions of network properties, such as the shortest path, Jaccard index, degree, clustering coefficient, and betweenness centrality, and used a supervised learning procedure (random neural network classifier) to model co-expression in terms of such scale-aware network metrics. They found that co-expression prediction improved when using multi-scale networks, suggesting that gene regulation and co-expression prediction benefits from using information from both direct chromatin interactions and indirect interactions occurring in a higher-order scale [127].

Malod-Dognin *et al*. analyzed the chromatin network structure of 17 healthy and one chronic lymphocytic leukemia cell lines using “graphlets” (small, connected, non-isomorphic motifs extracted from larger networks). In this study, data from CHi-C experiments was used, and nodes represented genes. The authors reported that leukemia cells show large network structural changes, with reduced modular organization and functional coherence. Additionally, driver genes became hubs that connect modules disconnected in normal cells. Moreover, both healthy and chronic lymphocytic leukemia cells were characterized by specific connectivity patterns in terms of “graphlet signatures”, that is, a signature distance called the “average graphlet degree vector distance” between the driver genes was smaller than the distances between background elements, which could be a useful finding to predict new cancer drivers in healthy cells [128].

More recently, we built the “Gene Regulation Graph Database” (**GREG**), a database of protein–protein, protein–DNA, DNA–DNA, ncRNA–DNA, and ncRNA–protein interactions for eight human cell lines, including three types of stem cells (H1, IPS6.9, and IPS19.11), four types of cancer cells (A549, K562, MCF-7, and HeLa), and a fibroblast cell line (IMR-90). In GREG, DNA bins, DNA-binding proteins, and DNA-binding ncRNAs are the nodes, while their interactions are the edges of the graph. Among other applications, we used GREG to predict transcription factories related to chronic obstructive pulmonary disease (COPD). First, we identified all GREG’s DNA bins containing the SNPs that have been correlated to COPD in lung fibroblasts (IMR-90 cells). Then, we extracted the networks of all protein, lncRNA, and DNA interactors in these bins. The results suggested that mutations in the CHRNA3, IL13, and MMP9 genes may affect chromatin hubs in chr15 (genes associated with effects of smoking), chr5 (genes associated with cytokine signaling, cell cycle, transport, and senescence), and chr20 (genes associated with immunity, inflammation, and transport), respectively. Finally, gene expression data for the genes in the identified hubs showed that several genes were downregulated in COPD, suggesting that the effects of COPD on lung fibroblasts are a consequence of the perturbation of such chromatin hubs/transcription factories [129].

### 3.3 Calling chromatin domains (TADs, LADs, NADs, etc.)

There are multiple computational methods for TAD prediction (see the review by Zufferey *et al.* [17]). Zufferey *et al.* compared 22 prediction methods (including network methods, clustering methods, linear score, and statistical models) and found that the reported TAD numbers and sizes vary among the different TAD callers. After comparing robustness, cost-effectiveness, concordance with other TAD callers, enrichment for biological features, and computational efficiency, the authors recommended TopDom [130], HiCseg [131], CaTCH [132], CHDF [133], and IC-Finder [134], as the best performing TAD callers.

The study also highlighted that most methods called large TADs when larger bin sizes were used, with the boundaries detected in these cases being a subset of the boundaries detected with smaller bin sizes. The authors interpreted this as support for TADs being organized in a hierarchical architecture of nested TADs, and thus, that the boundaries, TAD size, and TAD number are relative to the data resolution.

A final observation about TAD prediction is that, except for CTCF and cohesin, the features that predict TADs are different from the ones that predict enhancer–promoter interactions [115].

With regard to repressive hubs, it has been suggested that the B compartment derived from Hi-C studies corresponds to the union of all the heterochromatin hubs, such as nuclear lamina LADs, nucleolar NADs, and centromeric PADs [24]. The genome’s A/B (active/repressive) compartments have been predicted by correlation to epigenetic marks. Fortin *et al*. have shown that A/B compartments can be predicted using DNA methylation microarray, DNase hypersensitivity, sc-ATAC sequencing, and sc-whole-genome bisulfite sequencing data [160].

LADs can be called from either ChIP-seq or DamID data. Regarding ChIPseq-data-based callers, it must be considered that lamin peaks can be described as broad and low-level enrichment; thus, it is difficult to find them using ChIP-seq peak callers for TFs, such as MACS (which are designed for narrow peaks and high-enrichment), or peak callers for histone marks, such as SICER (which are designed for broad peaks, but not as broad as LAD peaks) [25]. Lund *et al.* introduced “enriched domain detector” (EDD), an algorithm to detect broad domains with low-level enrichment, such as heterochromatin regions. EDD is based on the idea that compact chromatin generates fewer sequence reads due to experimental difficulties, owing to which the number of reads is insufficient to detect enrichment. Instead, EDD compares sample read counts to input read counts, computing the ratio of sample reads to “sample + input” reads. The algorithm distinguishes between enriched informative, depleted informative, and non-informative bins, and significant clusters of enriched informative bins are identified as peaks [135]. Regarding DamID-data-based callers, Reddy *et al.* introduced LADetector [25], [136]. The software requires a bed file of log_2_ ratios of sample and control files, uses the “DNAcopy” package for data segmentation, and creates clusters between consecutive bins [136].

Although R-loops and G-quadruplexes may co-localize, computational tools have been developed to predict each of them. A recent prediction tool for R-loops is “R-Loop Tracker”, a web platform for R-Loop prediction and analysis from genomic DNA [137].

Different tools for prediction of G-quadruplex sequences [also called putative quadruplex sequences (PQSs)] have already been reviewed [161], [162]. Kwok *et al.* distinguished tools predicting “intramolecular G4s” (those formed from a single DNA strand) from tools predicting “intermolecular G4s” (from more than one DNA strand or a DNA–RNA hybrid). Intramolecular G4 prediction tools include tools such as Quad-Parser [138], QGRS Mapper [139], G4P Calculator [140], QuadBase [141], and G4Hunter [142]. Most of these tools are based on finding sequences with four tracks of three guanines in close proximity, originally the motif G_3_-N_1-7_-G_3_-N_1-7_-G_3_-N_1-7_-G_3_ (where N represents A, T, G, or C); however, several exceptions and different structures have been reported, and additional factors involved in G4 folding have been considered in the more recent algorithms [161]. Intermolecular prediction tools for both DNA strands include ddiQFP [143] and Allquads [144]. Methods such as ChIP-seq detect around 10,000 G4s, while G4-seq detects around 700,000. Most G4 prediction methods usually predict something in the middle, which has been interpreted as a need to consider more non-canonical G4 motifs [161].

### 3.4 Chromatin hub and transcription factory prediction methods

Multiple methods predicting chromatin hubs and transcription factories have emerged in recent years. In 2013, Ben-Elazar *et al*. published a statistical test to determine the existence of transcription factories. They examined whether co-regulated genes were clustered in space significantly more than their clustering along the genome, which proved to be true for 64 out of 117 TFs, *i.e.*, 64 types of specialized transcription factories, in *S.cerevisiae* [145].

Waszak *et al.* introduced the concept of “variable chromatin modules” (VCMs). After comparing expression, three histone marks, the TF PU.1, and RNAPol2 levels in lymphoblastoid cell lines from 47 individuals, they found that inter-individual variation was concentrated on chromatin modules that they termed VCMs and that TADs were enriched in such VCMs. They described VCMs as sub-TAD transcription-related spatial structures, similar to transcription factories [146]. Dai *et al.* used 3D structures of both deconvoluted Hi-C data and sc-HiC data to identify a series of 3D chromatin clusters that occurred frequently across the population. They called these clusters “regulatory communities,” as a large number of them were enriched on regulatory factors, with the two major factors being “centromere clustering” and “transcription factor binding” [147].

Capurso *et al.* superposed ChIP-seq data to a chromatin interaction network to detect 3D regions for which the ChIP-seq peak height was significantly high, which they called “functional 3D hotspots” and suggested that transcription factories could be one example of such hotspots [149]. In the same year, Zhu *et al.* introduced “EpiTensor,” a tensor modeling method to identify 3D spatial associations from 1D epigenomic information of histone marks, DNA accessibility, and gene expression. Using this method, the authors identified a group of “interaction hotspots” enriched on TFs and ncRNAs, which could be transcription factories [151].

Belyaeva *et al.* introduced a method to locate co-localized and co-regulated chromatin regions [150]. They built a graph with DNA ranges as bins and Hi-C interactions as edges, and added data on 48 TFs and histone modifications. They then assigned weights to the edges as correlations between epigenetic marks and performed weighted clustering. Finally, they used an eXtreme gradient boosting trees classifier to discover that clusters can be classified as either enriched on TFs and RNAPol2 (transcription factories) or enriched on H3K9me3 (repressive). 3D-FISH validation showed that RNAPol2 is indeed enriched in predicted factories. Stevens *et al.* performed a similar analysis for sc-HiC data [96]. After mapping ChIP-seq data onto the single-cell 3D networks, the authors found spatial clustering of histones H3K4me1, H3K27ac, and H3K4me3, which correspond to enhancer–promoter spatial clusters, corroborating that such clusters are not a mere artifact of bulk Hi-C studies.

Chromatin Module Inference on Trees (CMINT) is a related method that finds the dynamic changes of chromatin modules [148]. Here, a chromatin module was defined as a set of genomic loci with the same chromatin state (combination of seven histone marks). The modules and extent to which they were shared between different cells were determined using a probabilistic clustering approach. The method also determines which genomic loci switch modules and how likely are genomic loci in general to switch modules, which they apply to the study of both iPSCs reprogramming and hematopoiesis.

### 3.5 Multi-way chromatin interaction data

The advent of multi-way interaction detection methods and datasets (see Supplementary Material 1) has inspired the development of associated bioinformatics methodologies. Beagrie *et al.* introduced “Statistical inference of co-segregation” (SLICE), the algorithm to process data obtained from GAM [108]. SLICE compares the co-segregation frequency in GAM nuclear slices to the background co-segregation frequency and computes a probability of interaction.

Kim *et al.* introduced “multiplex interaction analysis by signal processing algorithms” (MIA-Sig) [152], a bioinformatics solution to remove noise and call significant chromatin complexes from ChIA-Drop data. The authors emphasize that the relationship between genomic distance and interaction probability is inversely proportional; therefore, multi-way algorithms have to reconcile this observation with having multiple genomic distances and interaction probabilities. MIA-Sig assumes that a genomic locus distant from the other loci in the droplet, should be interpreted as an error. The software contains modules to (i) remove experimental noise and call significant hubs, (ii) call TADs, and (iii) identify inter-TAD interactions, in multi-way data. The authors of Pore-C have also released the “Pore-C pipeline” to extract multi-way chromatin interactions from Pore-C concatemers [107].

“Multiway-interacting chromatin analysis” (MATCHA) is an example of a method that can be used to process multiple types of multi-way data [153]. MATCHA is an algorithm to study multi-way interaction data by using a hypergraph representation (multi-way interactions are represented as hyperedges). The authors criticize the assumptions of software such as MIA-Sig, by pointing out that a denoising strategy based on frequencies alone is problematic because the frequencies of hubs with a large number of loci are much smaller than that of small hubs. MATCHA was applied to both SPRITE and ChIA-Drop data to denoise the data and make *de novo* predictions. For SPRITE data, MATCHA enhanced the data quality by calling 700 k 3-way, 800 k 4-way, and 700 k 5-way interactions. Regarding prediction, the authors used their own hypergraph representation learning model, which uses labeled data and node features as input. MATCHA is used to distinguish multi-way interaction data from cliques of pairwise interaction data in a cell population.

Finally, Liu *et al.* presented a method for multi-way interaction prediction based on pairwise interaction frequencies from bulk Hi-C data [154]. The authors derived analytic expressions from polymer physics for n-body contact probabilities among chromatin loci, which are based on pairwise interaction frequencies. The equations allowed them to derive multi-way interaction probability maps and report that their predicted 3-body interaction probabilities agree with TriC, MC-4C, and SPRITE measurements.

### 3.6 A guide through the main computational approaches for chromatin hub studies using the GREG platform

Network analysis and machine learning are arguably the most common computational approaches to study chromatin hubs. To build a network or machine learning analysis, several aspects have to be considered. In Supplementary Material 2, we have included a summary and some links to computational tools and step-by-step open workflows that we have built to illustrate such basic steps and challenges.

## 4 Chromatin hubs: Towards a unified model

**Fig. 3:**
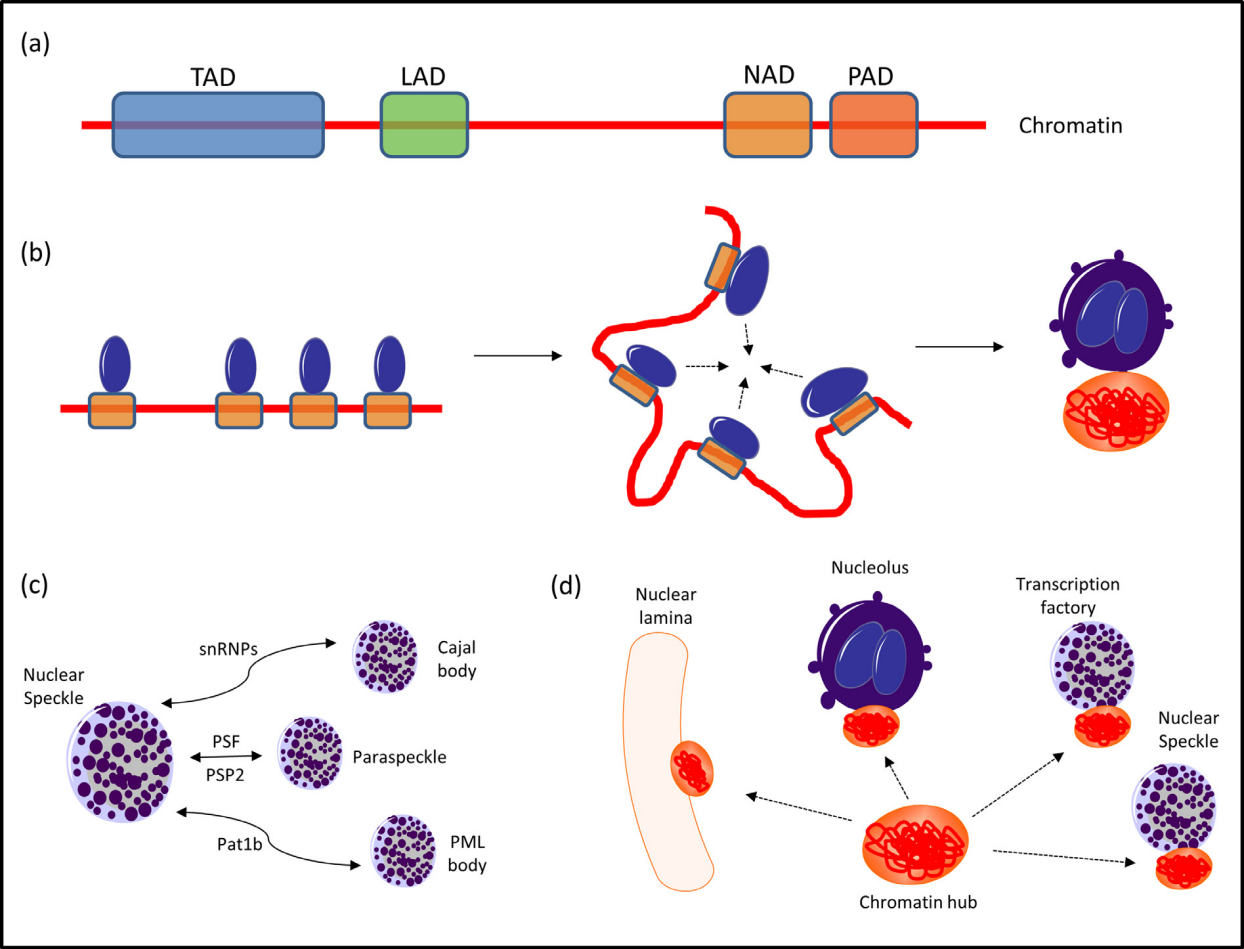
Some key research areas towards a unified model of chromatin hubs. (a) Identification of chromatin domains able to form chromatin hubs. In the figure, hypothetical TADs, LADs, NADs, and PADs are identified. (b) Nuclear body/chromatin hub biogenesis. In the figure, we show a model where liquid–liquid phase separation occurs near NADs forming small nucleoli, which then coalesce, generating a large nucleolus with an associated chromatin hub. (c) Common and distinct biological pathways in nuclear bodies and their effect on both organelle identity and chromatin binding. In the figure, nuclear speckles are shown to share different proteins with other nuclear bodies. (d) Chromatin domain potential for joining different repressive or active compartments and oscillating between them. In the figure, a hypothetical chromatin domain shows the potential to bind to four different nuclear structures.

We are starting to find the pieces of the puzzle to build a unified theory able to explain all of the different types of chromatin hubs (Fig. 3). Such pieces include (i) the existence of chromatin domains with the potential of generating interacting hubs (TADs, LADs, NADs, PADs, etc.), (ii) the assembly of chromatin hubs in space through a liquid–liquid phase-separation/compartmentalization process, (iii) the signature composition of such compartments (proteins, lncRNAs, *etc.*), and their similarities and differences to other compartments, (iv) the chromatin domain’s potential for stochastically joining different transcriptional or repressive environments/compartments, and (v) the potential of some chromatin domains for moving from transcriptional to repressive compartments (bi-stability).

### 4.1 A unified biophysical model

The liquid–liquid phase separation model has quickly become the strongest contestant to explain chromatin hubs [11]. Under such model, chromatin domains seem to hub around liquid compartments. The model explains organelle biogenesis as the coalescence of bubbles, while explains interaction specificity through such bubbles behaving as mini-cells containing receptor-like molecules at their surface (which for TFs means that there is no need of scanning all possible binding sites in a long DNA chain).

It has also been proposed that the interfaces of membrane-less compartments could have functional roles. Liao *et al.* put forward a model for splicing in nuclear speckles in which exons are transported into the speckles by SR proteins, while introns are barred entry by nucleoplasmic hnRNP proteins, leaving splicing sites at the nuclear speckle interface, where interface-located spliceosomes will perform the splicing reaction. The authors suggested that a similar organization of reactions could exist in other nuclear bodies, such as RNAPol1 factories/nucleoli [163].

The new model also brings to light new questions, *e.g.*, the processes involved in the genesis of the condensates. Recently, Qi *et al.* built a molecular dynamics simulation model where chromatin was represented as coarse-grained particles (either A compartments, B compartments, centromeric regions, or NADs) and suggested that there are two ways in which nucleolar particles associate with NADs and each other: In the absence of the chromatin network, all nucleolar particles condense into a single droplet, while, in the presence of the chromatin network, they condense into multiple droplets [164]. Another important question is whether the changes in transcriptional activation are directly related to phase separation. A recent study suggested that TF phase separation at promoters does not enhance transcription but is either neutral or inhibitive. Additionally, the mechanisms for transcription initiation, elongation, and termination between condensates remain unclear [32].

### 4.2 A unified mechanistic model

There is evidence of similarity in composition between many phase-separated compartments containing chromatin hubs, as well as evidence of protein exchange between them. For example, nuclear speckles share proteins with other types of nuclear bodies, such as (i) spliceosomal snRNPs with Cajal bodies, (ii) PSF and PSP2 with paraspeckles, and (iii) Pat1b with PML bodies [77]. Both cooperative and competitive interactions are found when studying specific inter-compartmental mechanisms. That suggests the idea of a spectrum of compartments instead of a few well-differentiated structures.

An early computational model of chromatin hubs was built for transcription factories and nuclear speckles. An important feature of nuclear speckles is that they form in the vicinity of RNAPol2 transcription sites. They contain several RNAPol2 subunits, are rich in proteins related to transcription elongation, and are poor in proteins related to transcription initiation [77], suggesting a complementary relationship between speckles and factories. Rieder *et al.* observed that co-expressed genes tend to be located in a “nuclear neighborhood” more often than by chance. Such nuclear neighborhoods are ∼ 1 μm in diameter and can be associated with either a factory or a speckle. The authors explained such organization with a mathematical model that reproduces the frequencies of speckles and factories sharing co-expressed genes as a stochastic selection process of a nuclear body (either factory or speckle) within a volume defined by the global organization existing before gene expression [79]. This model suggests that factories and speckles might have a redundant role.

Steensel *et al.* went one step further and introduced a model in which some chromatin regions (LADs) were linked to the nuclear lamina while others (inter-LADs) were linked to either transcription factories or nuclear speckles. The authors postulated that, similar to factories and speckles, some LADs can stochastically interact with the nuclear lamina, nucleolus, or centromeres, which correspond to three distinct repressive environments. As previously mentioned, LADs partially overlap with NADs; moreover, some NADs have been found near the nuclear lamina by FISH, while some LADs have been detected close to pericentromeric heterochromatin by 4C. Consequently, the model suggests that large heterochromatin domains are “LADs” if they interact with the nuclear lamina, “NADs” if they interact with the nucleolus, or “PADs” if they interact with centromeres; however, it is important to clarify that the three types of domains are similar but not identical, and some genomic regions show a preference for one over the others [24], [165].

The authors also introduced a model where a subset of LADs can oscillate between the nuclear lamina and either a transcription factory or a splicing speckle, alternating between being transcriptionally repressive and transcriptionally active. They suggest that the contact between the LADs and the nuclear lamina constitutes a separate 3D compartment, keeping them isolated from transcription factories or speckles. Therefore, a possible mechanism for LADs to move away from the lamina starts with active promoters at the LAD borders interacting with transcription factories or nuclear speckles and, as a consequence, the factories/speckles pull the edges of the LAD away from the nuclear lamina, bringing the entire domain with them [24].

More examples of such bi-stability have been reported. Zhao *et al.* have shown that during daytime, circadian genes are repressed through a CTCF- and PARP1- mediated recruitment to the nuclear membrane, which changes (activates) during night-time. Consequently, an oscillation of circadian genes occurs between active circadian loci and repressive LADs [166]. Computational studies have also shown the formation of assemblies or cliques of TADs, and the translocation of such assemblies to the nuclear periphery during differentiation, in a kind of TAD-to-LAD transition [167].

None of the previous models considers all of the known chromatin hubs. We suggest that a unified model should include three components: (i) the network of interactions and pathways for most transcription-related hubs (*e.g.*, factories, nuclear speckles, Trx domains, MALAT1 lncRNA, nascent RNAs, R-loops, G4s, and transcriptional histone marks), (ii) the network of interactions and pathways for most repression-related hubs (*e.g.*, LADs, NADs, PADs, and repressive histone marks), and (iii) the network of interactions and pathways for hubs with the potential of being bistable (*i.e.*, LADs becoming factories, PcG domains switching to Trx domains, *etc.*). Such networks might have a stochastic nature (*i.e.*, stochastic choice of interaction partners).

It is important to note that studies using sc-Hi-C suggest that not all of the abovementioned chromatin hubs have the same relevance. Stevens *et al.* reported that the organization of A/B compartments, LADs, and active enhancers and promoters, is consistent between single cells, while TADs and individual chromatin loops vary considerably from cell to cell [96].

### 4.3 A roadmap to build computational tools under the unified chromatin hub model

In terms of mapping known biological hubs to predicted computational hubs, the results are disappointing. To our knowledge, a comparison of computational methodologies against benchmark data does not exist. Moreover, computational methods do not go beyond predicting active *versus* repressive hubs, and therefore, do not reach the level of detail known in biology. Consequently, one of the immediate challenges is to generate hub-type-specific prediction algorithms, which would be important because different hubs have different functions. In principle, this would mean gathering data on the signatures summarized in Table 1 and mapping such data to our chromatin hub models to predict specific types of hubs. However, such a task presents several obstacles.

First, we must take into account the technology that will be used to generate the chromatin interaction datasets. Proximity-ligation methods are more conservative and only register hubs in close proximity, while ligation-free methods can detect hubs around nuclear bodies but might also introduce new artifacts. Thus, we must clarify which technologies are suitable to predict specific types of hubs. As an example, Arrastia *et al.* have stated that hubs around nuclear speckles can be detected by both sc-HiC and sc-SPRITE, while *peri*-nucleolar and *peri*-centromeric hubs can only be detected by sc-SPRITE [18]. A map of all chromatin interaction detection technologies *versus* all the hubs that they can potentially detect would be highly desirable. Multiple technologies should converge to address such problems, including (i) the development of more complex and specific signatures, (ii) the use of multiplexed imaging techniques, and (iii) the development of new experimental methods to detect chromatin in their specific hub/compartment.

A few technical problems must also be addressed. For example, many of the computational methods here reviewed still use hg19 data (see JEME’s and SEPT’s articles), a reference genome version that does not include centromere and other interesting heterochromatic data [168]. Therefore, updates to the most recent reference genome versions are compulsory for progress in the field.

## 5 Summary and outlook

In this review, we have built a comprehensive and unified model of the current progress in chromatin biology under the concept of a “chromatin hub.” The biological review involved the study of chromatin hubs made of chromatin–chromatin interactions only; hubs at the nuclear periphery; hubs around macromolecules, such as RNA polymerase, lncRNAs, and nascent RNAs; and hubs around nuclear bodies, such as the nucleolus or nuclear speckles (Section 2). The computational review included enhancer–promoter interaction prediction, network analysis methods, chromatin domain callers, transcription factory predictors, and multi-way interaction analysis (Section 3). We also discussed the elements of an integrated model in Section 4.

There are multiple reasons to study chromatin hubs. We believe that chromatin hubs may help explain:

(i) Gene regulatory networks and the redundancy of transcriptional regulators
(ii) The stochastic switch of interaction partners, and thus mechanisms, without changing the genome structure
(iii) DNA–DNA, protein–DNA, and lncRNA–DNA interaction specificity
(iv) New quantitative predictions, such as kinetic models in bubbles (*versus* models assuming diffusion towards the DNA molecule) or chromatin hub-aware gene set analysis
(v) Understanding disease: A hub-centric view could allow us to see why some perturbations are not dangerous (redundant mechanisms) while others disrupt the whole hub and lead to disease.

There are recent examples of mathematical modeling having into account chromatin hubs. Zuin *et al.* positioned enhancers at different distances of a promoter and observed that the contact probabilities between enhancer and promoter decay with increasing genomic distance, falling significantly when approaching the TAD boundaries and falling even further across the TAD boundary. A similar pattern is followed by the promoter's transcriptional output, where transcriptional levels decrease with increasing genomic distance and fall to promoter-only levels when the enhancer is located outside of the TAD and is not able to activate the promoter. Therefore, the authors built a mathematical model to explain transcriptional output in terms of contact probabilities: Transcription is described by a two-state (on–off) model where the frequency of transcriptional bursts depends on the enhancer-promoter contact probability through a Hill function [169].

Previously, we have reviewed how diseases attributed to a given gene may be related to distant genes thanks to long-range interactions [1]. In the same way, TAD disruption has been associated with cancer and other diseases [170], [171], [172]. LAD disruption has been associated with multiple muscular disorders [87]. Nucleolus-related diseases include ribosomal, cardiovascular, and neurodegenerative diseases; also, tumors and viruses that hijack the nucleolus to use it for either growth or viral replication [173]. Nuclear speckle-related disorders also include cancer, viral diseases, and neurological disorders. Overexpression of some speckle proteins, such as SR proteins, is observed in many types of cancer. Viral infection affects the localization and levels of splicing factors, and neurodegenerative diseases disrupt the nuclear speckles. Some rare disorders, such as retinitis pigmentosa, are generated by mutations in genes that encode speckle proteins or ncRNAs [77]. A few rare diseases have also been mapped to PML bodies [12]. Studies using an *in vivo* loss-of-function mouse model have revealed that CTCF depletion is enough to induce heart failure. Also, heart failure genomes display a decreased stability of chromatin interactions around cardiac disease genes [174]. One bioinformatics platform that interprets chromosomal-rearrangement-associated diseases in terms of disruption of chromatin structures is “3Disease Browser” [175]. We have also mentioned our study linking predicted chromatin hubs to COPD [129].

The study of chromatin hubs is a new challenge for chromatin biology and bioinformatics, and it is a path to explore the answers to new interesting questions, including: (i) Where in the nucleus are chromatin interactions occurring? (ii) What are the functional consequences of this? (iii) What are the processes leading to chromatin hub disruption? (iv) What are the processes occurring after chromatin hub disruption? (v) Why are chromatin hubs occurring in some individual cells but not in others? (vi) How can this knowledge be used for disease understanding and treatment? Answering such questions might become a path to a more realistic view of the genome, its regulatory processes, and their disruption.

## Author contributions

AM contributed to sections 1, 2, 3, 4, 5, and Supp.Mat. XWH and SJ contributed to section 3 and Supp.Mat. QJ and XL contributed to sections 2, 4, and 5. All authors have read and agreed to the final version of the manuscript.

## Funding

AM was funded by Guangzhou Medical University, high-level talent fund; SJ was funded by China Postdoctoral Science Foundation grant 2019M652847; QJ is supported by National Natural Science Foundation of China (NSFC) grants 82070983 and 81870679; XL is supported by the State Key Laboratory of Ophthalmology, Zhongshan Ophthalmic Center, Sun Yat-sen University, Guangzhou Scientific Research Plan 202102010179 and NSFC grant 82150710555.

## Declaration of Competing Interest

The authors declare that they have no known competing financial interests or personal relationships that could have appeared to influence the work reported in this paper.

## Data Availability

All data used in the supplementary material (GREG’s chromatin hubs) can be found at https://zenodo.org/record/6339915.

## Glossary

**Technologies**
3C: Chromosome conformation capture
4C: Chromosome conformation capture on chip / Circular chromosome conformation capture
CAGE: Cap analysis of gene expression
CCC: We use this acronym in the paper to refer to all technologies derived from chromosome conformation capture, not only the 3C technology
ChIA-PET: Chromatin interaction analysis by paired-end tag sequencing
ChIA-Drop: Droplet-based chromatin interaction analysis
C-HiC, EC-HiC: Promoter capture Hi-C, Enhancer capture Hi-C
ChIP-seq: Chromatin immunoprecipitation sequencing
DamID: DNA adenine methyltransferase identification
Hi-C, sc-HiC: High-throughput chromosome conformation capture, single-cell high-throughput chromosome conformation capture
SPRITE, sc-SPRITE: Split-pool recognition of interactions by tag extension, single-cell split-pool recognition of interactions by tag extension

**Biomolecules**
eRNA: Enhancer RNA
lncRNA: Long non-coding RNA
miRNA: Micro RNA
ncRNA: Non-coding RNA
PcG: Polycomb group
pre-mRNA: Pre–messenger RNA
rDNA, rRNA: Ribosomal DNA, ribosomal RNA
snRNA: Small nuclear RNA
TF: Transcription factor
tRNA: Transfer RNA
Trx: Trithorax group

**Chromatin domains**
G4s: G-quadruplexes
LADs: Lamina associated domains
NADs: Nucleolus associated domains
PADs: Pericentromeric associated domains
TADs: Topologically associating domains

**Others**
ChIN: Chromatin Interaction Network
COPD: Chronic Obstructive Pulmonary Disease
ESCs: Embryonic Stem Cells

## Appendix A Supplementary data

Supplementary Data 1 Supplementary Material 1 contains additional information not included in the main text due to lack of space.

Supplementary Data 2 Supplementary Material 2 contains a guide to the main approaches to study chromatin hubs using the GREG platform.
