# Supplementary material for: Chromatin Hubs: A biological and computational outlook
Source: Comput Struct Biotechnol J. 2022 Jul 5;20:3796–813. doi: 10.1016/j.csbj.2022.07.002 (PMC9304431; doi:10.1016/j.csbj.2022.07.002)
Supplement: Supplementary Data 1 — Supplementary Material 1 contains additional information not included in the main text due to lack of space. [file mmc1.docx]

Review

Chromatin Hubs: A biological and computational outlook

Antonio Mora^1,^*, Xiaowei Huang^1^, Shaurya Jauhari^1^, Qin Jiang^2^, and Xuri Li^3,^*

^1^ Joint School of Life Sciences, Guangzhou Medical University and Guangzhou Institutes of Biomedicine and Health (Chinese Academy of Sciences), Guangzhou 511436, P.R. China

^2^ Affiliated Eye Hospital of Nanjing Medical University, Nanjing 210000, P.R. China

^3^ State Key Laboratory of Ophthalmology, Zhongshan Ophthalmic Center, Sun Yat-Sen University, and Guangdong Provincial Key Laboratory of Ophthalmology and Visual Science, Guangzhou 510060, P.R. China

* Corresponding authors: A.M.: antoniocmora@gzhmu.edu.cn; X.L.: lixr6@mail.sysu.edu.cn

**Supplementary Material 1**

The following is additional content not included in the main text due to lack of space.

**Contents:**

1. Biological Review
   1. Review of experimental technologies to detect multi-way interactions
2. Computational Review
   1. Review of enhancer-promoter interaction prediction using sequence-based methods

**1. Biological Review**

**1.1. Review of experimental technologies to detect multi-way interactions**

**Table SM1.** Multi-way interaction detection methods.

| Method | Ligation-free | Single-cell | Long-read | Reference |
| --- | --- | --- | --- | --- |
| TM3C | No | No | No | [1] |
| C-walks | No | No | No | [2] |
| GAM | Yes | No | No | [3] |
| Multiplexed super-resolution FISH | No | Yes | No | [4] |
| Tri-C | No | No | No | [5] |
| MC-4C | No | No | Yes | [6] |
| SPRITE | Yes | No | No | [7] |
| ChIA-Drop | Yes | Yes | No | [8] |
| Pore-C | No | No | Yes | [9] |
| sc-SPRITE | Yes | Yes | No | [10] |

Ay *et al.* [1] developed “tethered multiple 3C” (TM3C), a protocol that allows identification of interactions in groups of three or four loci. Using two different libraries of the KBM7 cell line, the authors found 13.8–14.8M pairwise interactions, 211–820k 3-way interactions, and 1.7–25k 4-way interactions.

Jiang *et al.* [11] introduced a modification to 4C-seq, a technology that finds all chromatin interactions for a given bait region (“viewpoint”). In this version, multi-loci interactions can be identified in addition to pairwise interactions. The authors used their method to show that interactions within a super-enhancer occurred simultaneously.

Olivares *et al.* introduced C-walks [2], a 3C-based method that fragments and barcodes large proximity-ligation DNA concatemers and then computationally assembles proximity chains of ligation junctions that they call “C-walks”. In an analysis of K562 and mESC cells, the authors proposed that chromosome organization is not driven by multi-way interactions but “nested, possibly dynamic, pairwise contacts.”

Beagrie *et al.* introduced “genome architecture mapping” (GAM), a method that captures all the chromatin regions found in slices of the nucleus [3]. The method identifies all genomic loci in a large number of thin nuclear slices gathered at random orientations; the co-occurrence of pairs of loci is used to infer proximity, and thus, to call chromatin interactions. In mESCs, GAM found an abundance of 3-way interactions across the genome, especially among super-enhancers and highly transcribed regions. The authors also report that GAM and Hi-C matrices are highly correlated.

Li *et al.* introduced OCEAN-C [12], a method that combines FAIRE-seq (for open chromatin) and Hi-C (for chromatin interactions) to detect open chromatin interactions only. They report that OCEAN-C peaks are hubs of chromatin interactions (active promoters and enhancers); besides that, ChIP-seq data showed that the peaks/hubs are bound by multiple DNA-binding proteins.

Bintu *et al.* introduced “multiplexed super-resolution FISH,” an imaging method that uses sequential rounds of fluorescence in situ hybridization (FISH) to generate a 3D image of chromatin regions (around 2.5 Mb) as connected 30 kb beads [4]. The authors reported “TAD-like” domains and the prevalence of 3-way interactions, which were conserved even after cohesin depletion.

Oudelaar *et al.* introduced TriC, which is a 3C method to study chromatin interactions at single alleles, including higher-order interactions. The authors reported that most reads contained pairwise interactions (~50%), followed by 3-way interactions (approximately one-third) and >3-way interactions (approximately one-sixth); they described the latter as “regulatory hubs” in compartmentalized domains, formed by multiple enhancers and promoters [5].

Allahyar *et al.* introduced “multi-contact 4C” (MC-4C), a 4C-based method using Nanopore sequencing [6]. The authors argue that high-throughput methods do not offer the coverage to study high-resolution interaction maps at individual genes; therefore, they used an extension of 4C, which builds high-resolution maps between a chromatin region and the genome. In MC-4C, concatemers are kept large and sequenced using long-read sequencing (Nanopore sequencing). Most of the concatemers have 3–5 interactions, with some reaching up to 10. The study showed that the elements of the β-globin super-enhancer form a hub of simultaneous interactions that can accommodate two genes at the same time.

Quinodoz *et al.* introduced “split-pool recognition of interactions by tag extension” (SPRITE) [7]. Instead of ligation, the authors used a split-pool strategy to barcode individual chromatin complexes. After cross-linking and chromatin fragmentation, a series of cross-linked complexes are generated, barcoded, and sequenced. Using SPRITE, the authors detected multi-way interactions and interactions between large distances, which they use to identify one inactive hub of inter-chromosomal interactions around the nucleolus and one active hub around nuclear speckles.

Zheng *et al.* introduced ChIA-Drop, a method that (similar to ChIA-PET) detects chromatin interactions mediated by specific proteins but, unlike ChIA-PET, has the ability to detect multi-way interactions [8]. ChIA-Drop does not use proximity ligation but takes advantage of microfluidics and droplet-based sequencing. Fragmented chromatin is fed to a microfluidics device, where each chromatin complex is directed to a droplet containing reagents for amplification and barcoding, which will be followed by sequencing. The authors reported fruit fly data where half of ChIA-Drop complexes (around 1.5M) contained three or more fragments, while a few (around 20k) may have hundreds. For those complexes with six or more fragments (around 170k), the authors report that they can be mapped to TADs. Additionally, promoter–promoter interactions were not as common as suggested by other methods. In an RNAPol2 ChIA-Drop assay, 80% of active chromatin complexes included only one promoter. Regarding the other 20%, the authors stated that multiple promoters support the idea of transcription factories, although the number seems smaller than expected. In such factories, they found two mechanisms of transcription coordination: “co-transcription,” where all the promoters are active, and “imbalanced-transcription,” where the weaker genes behave as enhancers of the stronger genes.

Recently, Ulahannan *et al.* introduced “Pore-C,” a method that links Hi-C with Nanopore sequencing of the concatemers [9]. The authors applied Pore-C to chromatin hub detection of histone bodies and A/B compartments in GM12878 cells, and claim that Pore-C has more than 12-fold enrichment of interactions of degree >3 compared to SPRITE.

Arrastia *et al.* introduced “single-cell SPRITE” (scSPRITE) [10], a split-and-pool barcoding strategy to tag DNA fragments in single cells. The authors argue that sc-HiC cannot capture super-long-range and higher-order interactions, such as chromatin hubs around nuclear bodies, due to the constraint of proximity ligation, which is not present in the SPRITE protocol. As a consequence, sc-SPRITE calls a hundred times more interactions (around 35M in sc-SPRITE versus 375k in sc-HiC) despite producing ten times less reads. The proportion of inter-chromosomal interactions is also higher in sc-SPRITE (around 54% *versus* 6% in sc-HiC), which the authors identify as a weakness of proximity-ligation methods. According to the authors, only sc-SPRITE could be used for single-cell studies of chromatin hubs around nuclear bodies. They also reported findings of chromosome territories, A/B compartments, and TADs in many single cells, as well as inter-chromosomal chromatin hubs around nucleoli, nuclear speckles, and centromeres. Regarding nucleoli, the authors studied NADs (rDNA clusters) in mESCs, which generated nucleolar chromatin hubs in agreement with both microscopy data and bulk SPRITE data. On the other hand, nuclear speckles in mESCs only displayed chromatin hubs on 34% of the cells. Regarding peri-centromeric heterochromatin, such regions were not mapped to the genome at the time of the study, so the authors studied the first 10 Mb of each chromosome. As a result, 49% of the cells were found to contain two of such regions in close proximity. It is important to note that ensemble sc-HiC cannot detect centromere and nucleolar interactions, but it can identify nuclear speckles [10].

**2. Computational Review**

- 1. ***Review of enhancer-promoter interaction prediction using sequence-based methods***

“Predicting enhancer-promoter interactions” (**PEP**) uses a feature extraction strategy called PEP-Word, which is based on transforming the DNA sequences of enhancers or promoters into features through the use of the word embedding model. The authors concatenated the features for enhancers and promoters and annotated the interactions as positive or negative; then, they trained a gradient tree boosting classifier that allows prediction [13]. **EP2vec** followed the steps of PEP-Word and extracts vector features from the sequence using an unsupervised deep learning method called Paragraph Vector, followed by the supervised training of a gradient boosted regression trees classifier [14].

“Sequence-based promoter-enhancer interaction with deep learning” (**SPEID**) uses a convolutional neural network (CNN) layer to learn features, followed by a recurrent neural network (RNN) layer to model sequential dependencies of features, as well as a dense layer, which is a linear classifier. SPEID results include previously reported DNA-binding proteins such as CTCF, SRF, JUND, SPI1, SP1, EBF1, and JUN, as well as newly reported motifs such as BCL11A, ZIC4, E2F3, and FOXK1. The authors claim better results than PEP and TargetFinder but highlight that their current framework is cell line-specific, and therefore, unable to determine features that are consistent across cell types [15]. Zhuang *et al.* introduced two innovations. First, they pointed out that a simple CNN performs slightly better than the CNN-RNN hybrid developed by SPEID. Second, they suggested the use of transfer learning (TL) to train the CNN using the data of all cell lines instead of training and testing each cell line individually. The TL strategy showed improved cell type-specific predictions, but still no improvement for across-cell-type predictions [16].

Finally, **SEPT** is a method that uses a similar CNN and TL strategy to predict interactions from new cell types: in this case, a CNN learns the features of enhancer and promoter sequences, and an adversarial neural network by gradient reversal layer (TL) reduces the cell-line specific features. SEPT is reportedly able to recognize enhancer–promoter interactions in a new cell type if the locations of enhancers and promoters are provided [17].

**References**

1. Ay, F., et al., *Identifying multi-locus chromatin contacts in human cells using tethered multiple 3C.* BMC Genomics, 2015. **16**: p. 121.

2. Olivares-Chauvet, P., et al., *Capturing pairwise and multi-way chromosomal conformations using chromosomal walks.* Nature, 2016. **540**(7632): p. 296-300.

3. Beagrie, R.A., et al., *Complex multi-enhancer contacts captured by genome architecture mapping.* Nature, 2017. **543**(7646): p. 519-524.

4. Bintu, B., et al., *Super-resolution chromatin tracing reveals domains and cooperative interactions in single cells.* Science, 2018. **362**(6413).

5. Oudelaar, A.M., et al., *Single-allele chromatin interactions identify regulatory hubs in dynamic compartmentalized domains.* Nat Genet, 2018. **50**(12): p. 1744-1751.

6. Allahyar, A., et al., *Enhancer hubs and loop collisions identified from single-allele topologies.* Nat Genet, 2018. **50**(8): p. 1151-1160.

7. Quinodoz, S.A., et al., *Higher-Order Inter-chromosomal Hubs Shape 3D Genome Organization in the Nucleus.* Cell, 2018. **174**(3): p. 744-757 e24.

8. Zheng, M., et al., *Multiplex chromatin interactions with single-molecule precision.* Nature, 2019. **566**(7745): p. 558-562.

9. Ulahannan, N., et al., *Nanopore sequencing of DNA concatemers reveals higher-order features of chromatin structure.* bioRxiv, 2019.

10. Arrastia, M.V., et al., *Single-cell measurement of higher-order 3D genome organization with scSPRITE.* Nat Biotechnol, 2022. **40**(1): p. 64-73.

11. Jiang, T., et al., *Identification of multi-loci hubs from 4C-seq demonstrates the functional importance of simultaneous interactions.* Nucleic Acids Res, 2016. **44**(18): p. 8714-8725.

12. Li, T., et al., *OCEAN-C: mapping hubs of open chromatin interactions across the genome reveals gene regulatory networks.* Genome Biol, 2018. **19**(1): p. 54.

13. Yang, Y., et al., *Exploiting sequence-based features for predicting enhancer-promoter interactions.* Bioinformatics, 2017. **33**(14): p. i252-i260.

14. Zeng, W., M. Wu, and R. Jiang, *Prediction of enhancer-promoter interactions via natural language processing.* BMC Genomics, 2018. **19**(Suppl 2): p. 84.

15. Singh, S., et al., *Predicting enhancer-promoter interaction from genomic sequence with deep neural networks.* Quant Biol, 2019. **7**(2): p. 122-137.

16. Zhuang, Z., X. Shen, and W. Pan, *A simple convolutional neural network for prediction of enhancer-promoter interactions with DNA sequence data.* Bioinformatics, 2019. **35**(17): p. 2899-2906.

17. Jing, F., S.W. Zhang, and S. Zhang, *Prediction of enhancer-promoter interactions using the cross-cell type information and domain adversarial neural network.* BMC Bioinformatics, 2020. **21**(1): p. 507.
