# Supplementary material for: Chromatin Hubs: A biological and computational outlook
Source: Comput Struct Biotechnol J. 2022 Jul 5;20:3796–813. doi: 10.1016/j.csbj.2022.07.002 (PMC9304431; doi:10.1016/j.csbj.2022.07.002)
Supplement: Supplementary Data 2 — Supplementary Material 2 contains a guide to the main approaches to study chromatin hubs using the GREG platform. [file mmc2.docx]

Review

Chromatin Hubs: A biological and computational outlook

Antonio Mora^1,^*, Xiaowei Huang^1^, Shaurya Jauhari^1^, Qin Jiang^2^, and Xuri Li^3,^*

^1^ Joint School of Life Sciences, Guangzhou Medical University and Guangzhou Institutes of Biomedicine and Health (Chinese Academy of Sciences), Guangzhou 511436, P.R. China

^2^ Affiliated Eye Hospital of Nanjing Medical University, Nanjing 210000, P.R. China

^3^ State Key Laboratory of Ophthalmology, Zhongshan Ophthalmic Center, Sun Yat-Sen University, and Guangdong Provincial Key Laboratory of Ophthalmology and Visual Science, Guangzhou 510060, P.R. China

* Corresponding authors: A.M.: antoniocmora@gzhmu.edu.cn; X.L.: lixr6@mail.sysu.edu.cn

**Supplementary Material 2**

**A guide to the main computational approaches for chromatin hub studies using the GREG platform**

Network Analysis (NA) and Machine Learning (ML) are arguably the two most common computational approaches to study chromatin hubs. In order to perform NA or ML analysis, several aspects must be taken into account. Here, we summarize these aspects and include some links to tools or step-by-step open workflows that we have built to illustrate the basic steps and challenges. All our examples use interaction data from the GREG database (http://www.moralab.science/GREG/).

Network Analysis

1. Nodes and edges can be defined in different ways depending on the aims of the study. One way to represent nodes is as DNA bins, *i.e.*, fixed segments of a chromosome with a specific size; a second model (gene-centered) only considers genes as nodes; and a third model (promoter–enhancer-centered) only represents promoters and enhancers as nodes. Choosing a DNA bin size is largely arbitrary, but it is also tied to the resolution of the chromatin interaction detection technologies. Regarding the definition of promoters and enhancers, there are also different alternatives: first, as a region around the TSSs (promoters) or p300 binding sites (enhancers); second, as experimentally verified promoters and enhancers; or third, as regions identified as promoters and enhancers by segmentation algorithms (*i.e.*, ML classifiers based on informative histone marks).

As an example of a chromatin network, we direct you to our online resource (http://www.moralab.science/GREG/), where nodes represent DNA bins, TFs, and lncRNAs. We have built two databases, one based on small bins (2 kb) and one based on large bins (200 kb). All interaction information (edges) has been adapted to our chosen bins.

1. The resulting network is dependent on the technology used. For example, ChIA-PET interactions are bulk and protein-mediated, while sc-HiC are single-cell and proximal (ligation-dependent). Therefore, we need to select the appropriate type of data for our analysis. In our example, GREG is an integrative database, which means that we have collected interaction data from multiple methods and added information about the method with which each interaction was obtained. Therefore, it is up to the users to filter only the interactions of their interest.

Another point to highlight is that the full network is difficult to visualize and we might be interested in specific subnetworks. With GREG, we can work on top of the chromatin network, moving nodes, deleting nodes, and expanding nodes to all their interactors; this way, we can build a unique subnetwork and obtain its statistics (http://www.moralab.science/GREG/).

1. Graph theory computations: there are several network analyses that can be performed by most graphical libraries in R, Python, or Cytoscape. For example, degree, centralities, shortest paths, and communities/modules. Other interesting network representations include hypergraphs and graphlets.

General statistics for the entire database tell us that the network had 2.78 M nodes and 19.4 M interactions (http://www.moralab.science/GREG/). In our platform, we have added degree and shortest path computations, as well as the option to download the subnetwork in a graphical format compatible with most graphical libraries, which allows the user to perform more complex analyses.

Defining hubs

Chromatin hubs can be defined in several different ways. In multiple studies, they are graph clusters or modules where intra-modular interactions are significantly more than inter-modular interactions. A second interpretation is that a hub is simply a node with a high degree, *i.e.*, a node with multiple interactions, regardless of whether they form a cluster. A third interpretation is a cluster created after giving weight to the nodes by, for example, adding the amount of ChIP-seq reads.

In our study, we defined “GREG’s chromatin hubs” as all DNA bins that contained more than one interaction (either protein–DNA, DNA–DNA, or ncRNA–DNA), but we restricted the list to only those hubs that had at least one DNA–DNA long-range interaction, to explore hubs with functional potential. GREG has two versions, one with 200-kb bins and one with 2-kb bins; we used the version with the small bins. A list of “GREG’s chromatin hubs” for our six cell lines can be found here: https://zenodo.org/record/6339915

Machine Learning

1. An ML classifier is used to identify the category that a given observation belongs to (in our case, whether a bin is either a “hub” or a “non-hub”). In practice, this means that nodes or groups of nodes should be labeled as hubs or non-hubs to train an algorithm, which will be used to predict the label of other nodes or groups of nodes.
2. The two basic elements of a classifier are the “classes” and the “features.”

In our example, we built different models to predict the “class” or “label” of a 2-kb DNA bin, which can be a “hub” or “non-hub” (https://github.com/mora-lab/GREG-Hubs/tree/main/MachineLearning/workflows).

The “features” are individual measurable variables that contain information about the classes, and therefore, help us building a better model. In our case, the abovementioned files (https://zenodo.org/record/6339915) also show the features that we used for each cell line. They are some of the features that have been identified in different hub prediction studies (*e.g.*, DNA-binding proteins and histone marks).

1. Multiple ML methods have been used, with Deep Learning methods being state-of-the-art. We have written several Jupyter notebooks to share the analysis workflow using different approaches and R packages, which can be found on our website (https://github.com/mora-lab/GREG-Hubs/tree/main/MachineLearning/workflows). We highlight two methodologies, namely, logistic regression and random forests, and we evaluate a couple of R packages for each. The same workflows were repeated for six cell lines. All the workflows, results, and conclusions can be found on the abovementioned website.
2. The class imbalance problem: imbalanced data refers to the case where classes are not equally represented. This is our case, where non-hubs (represented as bins) are much more common than hubs. There are multiple ways to address the class imbalance problem, such as resampling the dataset or generating synthetic samples. We have addressed this problem in our Jupyter notebooks, where we reviewed a few methods before selecting SMOTE (Synthetic minority oversampling technique). A detailed explanation can be found on the abovementioned website.
